# ECG Utilization Patterns of Patients With Arrhythmias During COVID-19 Epidemic and Post-SARS-CoV-2 Eras in Shanghai, China

**DOI:** 10.3389/fcvm.2022.829679

**Published:** 2022-04-27

**Authors:** Cheng Li, Mu Chen, Mohan Li, Haicheng Wang, Xiangjun Qiu, Xiaoliang Hu, Qunshan Wang, Jian Sun, Mei Yang, Yuling Zhu, Peng Liao, Baohong Zhou, Min Chen, Xia Liu, Yuelin Zhao, Mingzhen Shen, Jinkang Huang, Li Luo, Hong Wu, Yi-Gang Li

**Affiliations:** ^1^Department of Cardiology, Xinhua Hospital, School of Medicine, Shanghai Jiao Tong University, Shanghai, China; ^2^Shanghai Siwei Medical Co. Ltd., Shanghai, China; ^3^Medical Information Telemonitoring Center, School of Medicine, Shanghai Jiao Tong University, Shanghai, China; ^4^School of Public Health, Fudan University, Shanghai, China; ^5^Shanghai Health Commission, Shanghai, China

**Keywords:** epidemic, COVID-19, health-seeking behavior, arrhythmias, atrial fibrillation

## Abstract

**Background:**

The COVID-19 pandemic has led to concerns around its subsequent impact on global health.

**Objective:**

To investigate the health-seeking behavior, reflected by ECG utilization patterns, of patients with non-COVID-19 diseases during and after COVID-19 epidemic.

**Methods:**

Taking advantage of the remote ECG system covering 278 medical institutions throughout Shanghai, the numbers of medical visits with ECG examinations during the lockdown (between January 23 and April 7, 2020), post-lockdown (between April 8 and December 31, 2020) and post-SARS-CoV-2 (between January 23 and April 7, 2021) periods were analyzed and compared against those during the same periods of the preceding years (2018 and 2019).

**Results:**

Compared with the same period during pre-COVID years, the number of medical visits decreased during the lockdown (a 38% reduction), followed by a rebound post-lockdown (a 17% increase) and a fall to the baseline level in post-SARS-CoV-2 period. The number of new COVID-19 cases announced on a given day significantly correlated negatively with the numbers of medical visits during the following 7 days. Medical visit dynamics differed for various arrhythmias. Whereas medical visits for sinus bradycardia exhibited a typical decrease-rebound-fallback pattern, medical visits for atrial fibrillation did not fall during the lockdown but did exhibit a subsequent increase during the post-lockdown period. By comparison, the volume for ventricular tachycardia remained constant throughout this entire period.

**Conclusion:**

The ECG utilization patterns of patients with arrhythmias exhibited a decrease-rebound-fallback pattern following the COVID-19 lockdowns. Medical visits for diseases with more severe symptoms were less influenced by the lockdowns, showing a resilient demand for healthcare.

## Introduction

The Coronavirus disease 2019 (COVID-19) is a highly contagious viral infection and has spread around the world by multiple transmission modes. (1) The outbreak of COVID-19, caused by SARS-CoV-2, led to an unprecedented global public health crisis, and resulted in a large death-toll and long-term side effects for the survivors of the disease. The Chinese government imposed lockdown measures in Wuhan, the epicenter of the outbreak, from January 23 to April 7, 2020, to fight against SARS-CoV-2 in China. Since March 11, 2020, the daily number of new confirmed cases of COVID-19 has decreased significantly in China. (2) With the epidemic under control, China's prevention and control strategy were gradually adjusted to facilitate the recovery of normal economic production and life in China (3–5).

Not only did patients with COVID-19 suffer serious health damage during epidemic, but the shortage of medical resources and the strict preventive measures necessary to deal with the outbreak have also posed challenges to the routine management of non-COVID-19 diseases such as cardiovascular diseases (6–8), which has been reported in a Danish Nationwide Cohort Study regarding all-cause mortality and location of death in patients with established cardiovascular disease in COVID-19 epidemic. (9) Previous studies, mostly based on large medical centers (10), showed that the number of hospital visits decreased during the lockdowns, but research on the health-seeking behavior of populations at large have been less reported. One study from Israel showed a decrease in hospital admissions for myocardial infarction during the early stage of the pandemic, as well as a rebounding increase as the first wave of the pandemic faded (11). This research focused, however, on one specific disease and does not reflect the health-seeking dynamics of other cardiovascular diseases. The current evidence focusing on the association between COVID-19 and cardiovascular diseases is based on small, disease-specific studies and lacks quantitative backing, which highlights the importance of our study regarding the health-seeking dynamics associated with various cardiovascular diseases during and following the epidemic.

As a widely adopted routine examination characterized by its easy access and low cost, electrocardiograms (ECG) act as the diagnostic method for various types of cardiac arrhythmias. Therefore, changes to the number of medical visits with ECG examinations might reflect changes in the medical-seeking behavior of patients with cardiac arrhythmias. Taking advantage of the largest remote ECG platform in China, we were able to glimpse how COVID-19 affected the health-seeking behavior of patients with various arrhythmias during and after the epidemic.

## Methods

### Study Population

**Figure 1:**
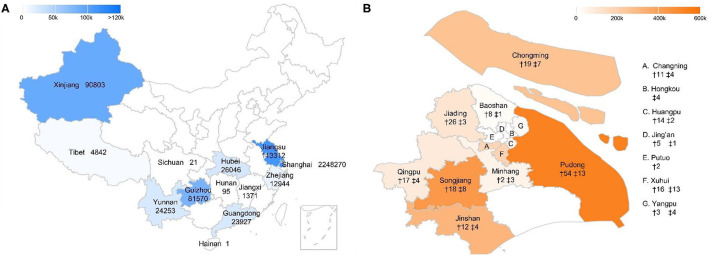
Medical institutions covered by the ECG platform in China and Shanghai. **(A)** Regions covered by the remote ECG platform in China. The number following the province name shows the number of medical visits with ECG examinations in that province between Jan 1, 2018 and Apr 7, 2021. **(B)** Number of medical institutions covered by the ECG platform in Shanghai. † and ‡ indicate the number of community clinics and academic hospitals in that district.

This study was approved by the ethics committee at Xinhua Hospital Affiliated to Shanghai Jiao Tong University School of Medicine. All data used for this research were derived from the Siwei (Shanghai Siwei Medical, Shanghai, China) remote ECG diagnosis system. (12) As the largest ECG diagnostic system in China, the platform collected ECG data from 1,320 medical institutions across 13 provinces in China, with a volume of more than one million medical visits involving ECGs per year (Figure 1A). As approximately 85% of this ECG data was collected from Shanghai (Supplementary Table 1), which covered 278 medical institutions from almost all administrative districts (Figure 1B and Supplementary Table 2), the present study analyzes ECG data in Shanghai between January 1, 2018 and April 7, 2021.

### Research Design

The numbers of medical visits with ECG examinations (ECG visits) during the lockdown (between January 23 and April 7, 2020), post-lockdown (between April 8 and December 31, 2020) and post-SARS-CoV-2 (between January 23 and April 7, 2021) periods were compared with those of the same periods during the two years prior to the COVID-19 outbreak (averages taken from 2018 and 2019). Subgroup analyses were then performed according to sex (male or female), age ( ≤ 59 years old, 60–79 years old or ≥ 80 years old) and the tier of medical institution (academic hospitals or community clinics).

The number of new COVID-19 cases, new deaths, new discharged cases and existing confirmed cases in the Chinese mainland were then analyzed based on daily updates from China's Center of Disease Control (CDC) between January 11, 2020, and April 7, 2021. (2, 13) Spearman's rank correlation was analyzed for the COVID-19 data and the number of medical visits concerning various arrhythmias.

### ECG-Based Diagnoses of Arrhythmias

ECG diagnoses, derived from a combination of artificial intelligence (AI) assistance and clinician diagnosis (details in Supplementary Methods), were adopted for the following diseases: sinus bradycardia, sinus tachycardia, atrial extrasystole, atrial tachycardia, atrial flutter, atrial fibrillation, ventricular extrasystole, ventricular tachycardia, paroxysmal supraventricular tachycardia(SVT), first-degree AV block (AVB), severe AVB (including second-degree type 2, high-degree and third-degree AVB), right bundle branch block (RBBB), left bundle branch block (LBBB) and left anterior fascicular block. Other abnormal ECG readings, such as myocardial infarction and ST segment depression, were not analyzed specifically for the inaccuracy of diagnosis due to lack of sufficient medical information.

### Statistical Analysis

The numbers of daily medical visits involving ECG examinations were regarded as continuous variables, and the Mann-Whitney non-parametric test was used to compare the changes in the number of ECG visits between the different time periods or subgroups. Chi-square tests were used to compare the differences in the proportions of various arrhythmias between academic hospitals and community clinics. Spearman correlation analyses were used to reveal the correlations between the number of COVID-19 cases and the number of medical visits on following days. Statistical analyses were performed using IBM SPSS Statistics 24 (SPSS Inc., Chicago, IL). A two-sided *p* value of < 0.05 was considered significant.

## Results

### Medical Visits During the Pre-COVID-19 Years (2018 and 2019)

The number of medical visits with ECG examinations remained stable during the two consecutive years preceding the COVID-19 pandemic. ECG visits in Shanghai were 696,800 and 702,989 in 2018 and 2019, respectively. Averages from 2018 and 2019 were taken as a pre-COVID baseline for subsequent comparison in this study.

**Figure 2:**
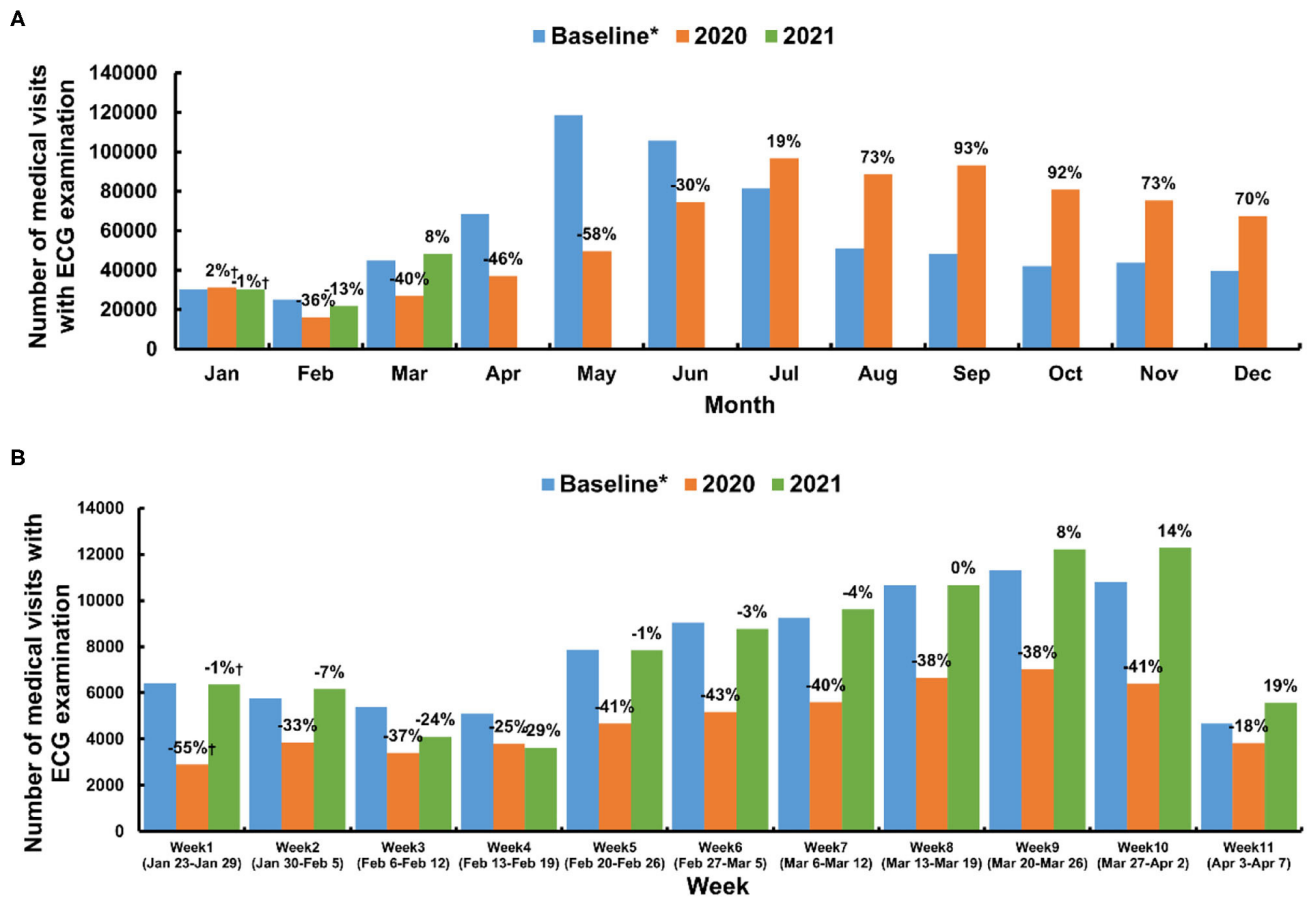
Medical visits with ECG examination in Shanghai. **(A)** Monthly medical visits with ECG examinations of the baseline, 2020, and 2021. **(B)** Weekly medical visits during the lockdown (between January 23 and April 7, 2020) and the same period from the preceding years (baseline) and 2021. *Average number taken from 2018 and 2019.†The percentage difference compared with the baseline.

The number of the ECG examinations were lower in the Jan-Feb and higher in May and June (Figure 2), different from the common sense that the cardiac disease patients are more prevalent in the Winter, the underlying reason remains unclear, and we checked the data from previous years. This pattern could also be observed in 2016, 2017, 2018, and 2019 (Supplementary Figure 1), future analysis is needed to clarify this issue.

### Medical Visits During and After the Epidemic

Table 1 shows that the number of medical visits with ECG examinations decreased from 86,232 to 53,246 (a 38% reduction) during the lockdowns, after which it then increased from 591,661 to 657,774 (an 11% increase) during the post-lockdown period. Finally, this figure returned to its baseline level during the post-SARS-CoV-2 period (87,178 compared to the baseline of 86,232). When compared with the same periods during pre-COVID years, there were fewer monthly medical visits between February and June 2020, an equal number in July 2020, and higher-than-baseline levels between August and December 2020 (Figure 2 and Supplementary Tables 3, 4). In addition, the yearly peak in the volume of medical visits occurred in May or June between 2016 and 2019, while in 2020, this peak was postponed to July (Supplementary Figure 1).

**Table 1:** Dynamics of medical visits of subgroups during different periods.

**Groups**	**All year round**			**Lockdown**			**Post-lockdown**			**Post-SARS-CoV-2**	
	Baseline *, n	**2020, n**	**Percentage change †**	Baseline *, n	**2020, n**	**Percentage change †**	Baseline *, n	**2020, n**	**Percentage change †**	**2021, n**	**Percentage change †**
Groups											
Males	321669	331239	3%	38834	23305	−40%	273130	295456	8%	40413	4%
Females	378226	407128	8%	47398	29941	−37%	318531	362318	14%	46765	−1%
≤ 59y	206975	214259	4%	32354	19285	−40%	165738	184151	11%	33855	5%
60-79y	389963	420944	8%	38971	23503	−40%	341967	386286	13%	40660	4%
≥80y	102957	103164	0%	14907	10458	−30%	83957	87337	4%	12663	−15%
Academic hospitals	139083	205164	48%	23273	22231	−4%	108788	171500	58%	30157	30%
Community clinics	560812	533203	−5%	62959	31015	−51%	482874	486274	1%	57021	−9%
Total	699895	738367	5%	86232	53246	−38%	591661	657774	11%	87178	1%

* *Average number of 2018 and 2019*.

† *Compared with baseline*.

### Relationship Between Medical Visits in Shanghai and Prevalence of COVID-19 in China

**Figure 3:**
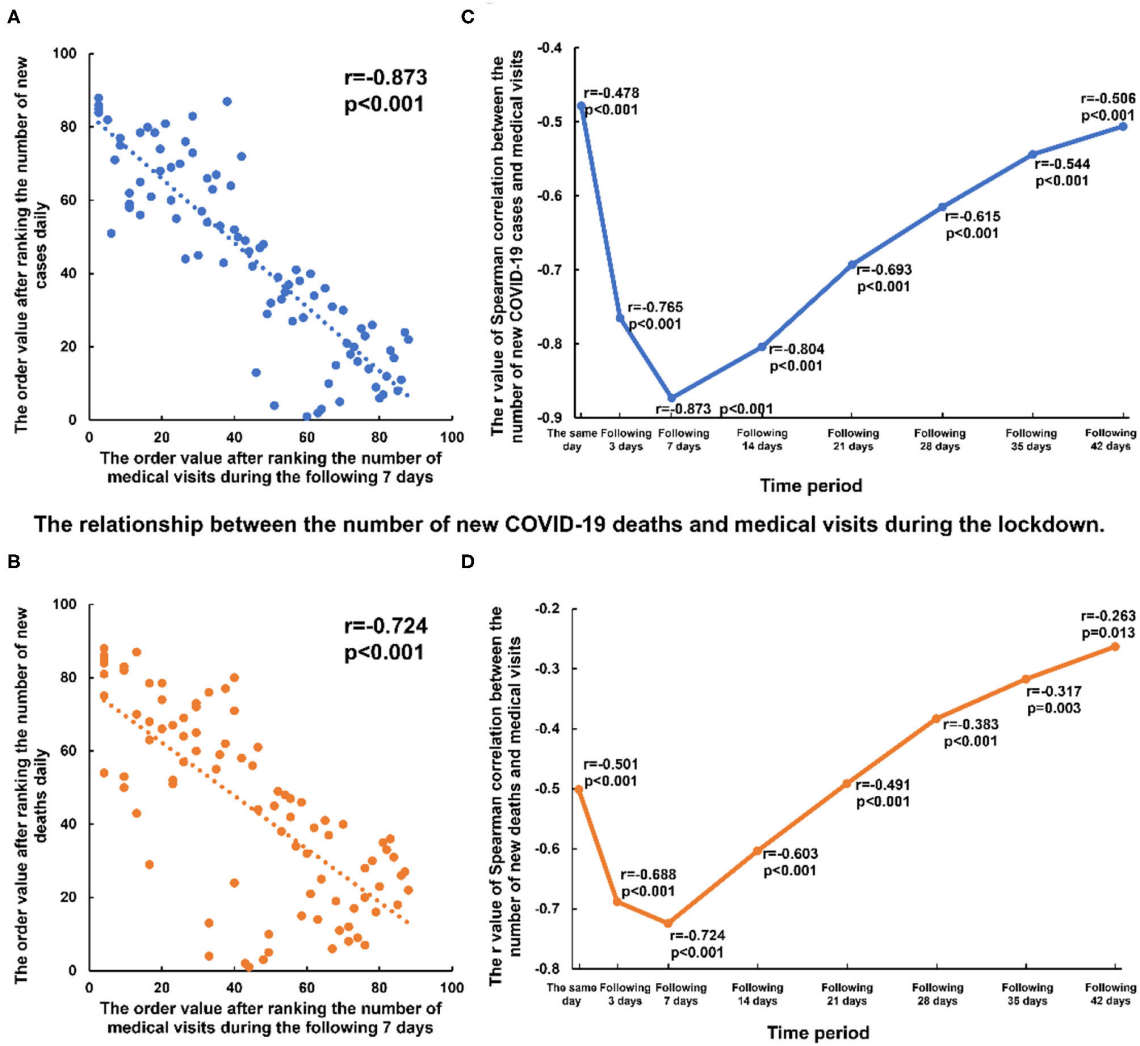
Correlation between the number of new COVID-19 cases or deaths and medical visits of the following days during the lockdown. **(A,B)** the negative correlation between the new COVID-19 cases or deaths and the number of medical visits with ECG examination of the following 7 days during the lockdown. **(C,D)** the correlation coefficient (r) between the new COVID-19 cases or deaths and the number of medical visits of the same day and the following 3–42 days.

We analyzed the relationship between the number of new confirmed cases per day during the lockdowns in Chinese mainland and the number of medical visits in Shanghai. During the lockdowns, the number of new confirmed cases per day was negatively correlated with the number of medical visits during the following three days (r = −0.765, p < 0.001), seven days (r = −0.873, *p* < 0.001), 14 days (r = −0.804, p < 0.001), 21 days (r = −0.693, *p* < 0.001), 28 days (r = −0.615, *p* < 0.001), 35 days (r = −0.544, *p* < 0.001) and 42 days (r = −0.506, *p* < 0.001) in Shanghai (Figure 3, Supplementary Figure 2 and Supplementary Table 5). This correlation coefficient exhibited a U-shaped curve, with a nadir at 7 days. Similarly, negative correlations with a U-shaped correlation coefficient curve were detected between the number of new COVID-related deaths in China and the number of medical visits in Shanghai during the lockdowns (Figure 3, Supplementary Figure 2 and Supplementary Table 5). Such a correlation was not detected between the number of new discharged cases or existing confirmed cases and medical visits in Shanghai during the lockdown (Supplementary Table 6). No correlations between medical visit in Shanghai and COVID-19 prevalence in China were detected during the post-lockdown and post-SARS-CoV-2 periods (Supplementary Table 5 and Supplementary Figure 3).

We further analyzed the relationship between medical visits in Shanghai and COVID-19 prevalence before and after the Spring Festival to exclude the impact of migration during the Spring Festival (January 23 and February 2, 2020). A similar negative correlation was observed even after factoring in for the Spring Festival migration (Supplementary Table 7).

### Subgroup Analyses of Medical Visits in Shanghai

**Figure 4:**
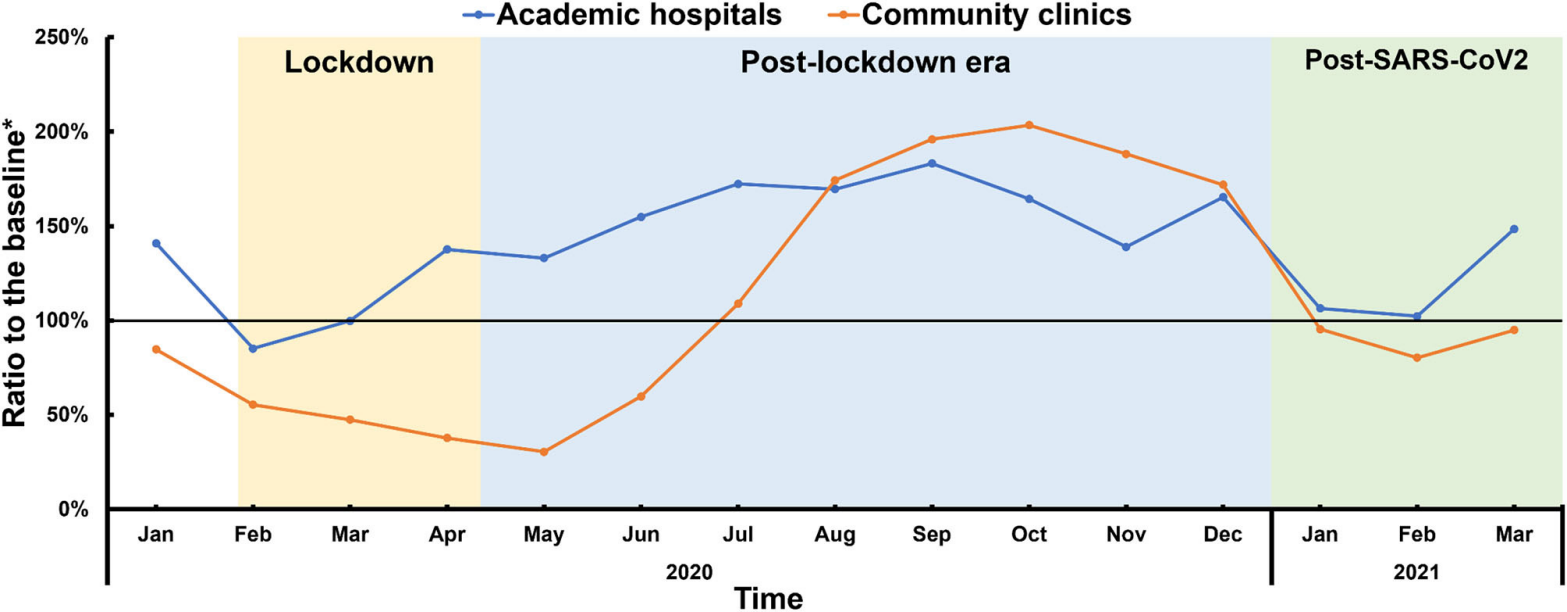
Medical visits to academic hospitals and community clinics. *Ratio to the average number of medical visits between 2018 and 2019.

The impact of COVID-19 on health-seeking behavior related to cardiac arrhythmias varied by tier of medical institution (Figure 4). Compared with the same period during pre-COVID years, the number of medical visits to academic hospitals did not decrease during the lockdowns but increased during the post-lockdown (a 58% increase) and post-SARS-CoV-2 periods (a 30% increase). ECG visits to community clinics, however, decreased drastically during the lockdowns (a 51% reduction) followed by rebounding growth post-lockdowns and a subsequent recovery to the baseline level during the post-SARS-CoV-2 period.

In addition, medical visits from different sexes and above the age of 60 years generally followed the same decrease-rebound-fallback pattern, which was most prominent in patients between 60 and 79 years old. Medical visits by patients under 60 years of age, however, remained at a relatively constant level throughout the entire period (Table 1, Supplementary Tables 3, 4 and Supplementary Figures 4, 5).

Similar to the results for the entire population, subgroup analyses based on age, sex and tier of medical institution revealed negative correlations between daily new cases or deaths with medical visits during the following 21 days during the lockdowns but not during the post-lockdown and post-SARS-CoV-2 periods (Supplementary Tables 5, 6).

### Disease-Specific Medical Visits During and Following the Epidemic

**Figure 5:**
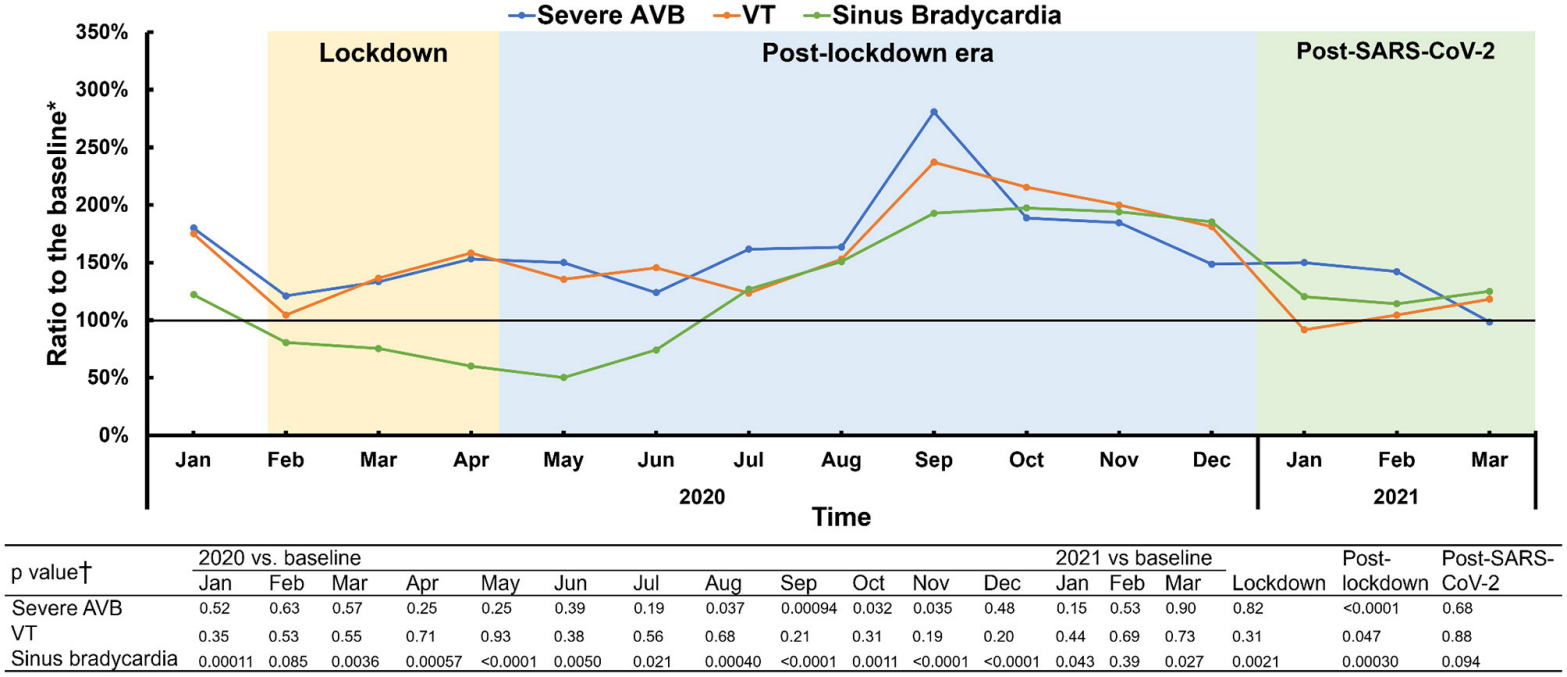
Different patterns of dynamics in disease-specific medical visits, represented by severe AVB, VT and sinus bradycardia. Three patterns of disease-specific medical visits dynamics during and following the pandemic were noticed, according to the ratio to the medical visits of the pre-COVID period. Pattern 1 (stable-increase-stable pattern) is represented by severe AVB. Pattern 2 (stable-stable-stable) is represented by ventricular tachycardia. Pattern 3 (decrease-rebound-fallback) is represented by sinus bradycardia. *Baseline was determined by the average number from 2018 and 2019. †The comparisons were obtained by a Mann-Whitney test. AVB, atrioventricular block; VT, ventricular tachycardia. The severe AVB includes second-degree type 2, high-degree and third-degree AVB.

The number of medical visits were further analyzed according to different arrhythmias during the lockdown, post-lockdown, and post-SARS-CoV-2 periods. During the lockdowns, the number of medical visits related to arrhythmias with severe symptoms, such as atrial flutter (*p* = 0.230), atrial fibrillation (*p* = 0.172) and severe AVB (*p* = 0.816) were comparatively similar with those from the same period during the pre-COVID years, revealing a high degree of rigidity in health seeking demand. By comparison, medical visits for diseases with little to no symptoms, such as sinus bradycardia (*p* = 0.002), ventricular extrasystole (*p* = 0.004) and RBBB (*p* = 0.001) significantly fell during the lockdown period. No matter the diagnosis, the number of medical visits exceeded baseline levels during the post-lockdown period, followed by a gradual fall to baseline levels during the post-SARS-CoV-2 period. Medical visits for ventricular tachycardia remained at a relatively constant level throughout the entire period analyzed (Figure 5, Table 2, Supplementary Table 8 and Supplementary Figures 6, 7).

**Table 2:** Dynamics of medical visits of various ECG events during different periods.

**Category of ECG events**	**All year round**	**Lockdown**	**Post-lockdown**	**Post-SARS-CoV-2**
	Baseline *, n	**2020, n**	**Percentage change †**	Baseline *, n	**2020, n**	**Percentage change †**	Baseline *, n	**2020, n**	**Percentage change †**	**2021, n**	**Percentage change †**
Normal ECG	310113	317929	3%	37617	20207	-46%	263332	286963	9%	37689	2%
Sinus bradycardia	64264	69573	8%	6393	4876	−24%	58051	64796	12%	7872	23%
Sinus tachycardia	28500	29462	3%	7075	5866	−17%	20126	22821	13%	5870	−17%
Atrial extrasystole	44217	45725	3%	6822	5167	−24%	36416	39526	9%	6249	−8%
Atrial tachycardia	3574	3840	7%	681	567	−17%	2779	3130	13%	570	−16%
Atrial flutter	1527	2204	44%	279	354	27%	1193	1802	51%	303	9%
Atrial fibrillation	20387	22060	8%	3466	3367	−3%	16334	18374	12%	3093	−11%
Ventricular extrasystole	26625	28982	9%	4371	3652	−16%	21667	24812	15%	4297	−2%
Ventricular tachycardia	141	206	47%	26	32	23%	110	168	53%	31	19%
Paroxysmal SVT	1168	1391	19%	203	231	14%	940	1167	24%	216	7%
First-degree AVB	25299	29582	17%	3188	2924	−8%	21664	26068	20%	3707	16%
Severe AVB	348	495	42%	61	78	28%	282	413	47%	72	18%
RBBB	36882	40705	10%	4890	4023	−18%	31337	35997	15%	5181	6%
LBBB	3309	3810	15%	476	427	−10%	2800	3324	19%	486	2%
Left anterior fascicular block	6863	6452	−6%	1000	692	−31%	5741	5656	−1%	904	−10%

* *Average number of 2018 and 2019*.

† *Compared with baseline*.

*SVT, supraventricular tachycardia; atrioventricular junctional tachycardia; AVB, atrioventricular block; RBBB, right bundle branch block; LBBB, left bundle branch block*.

*The severe AVB includes second-degree type 2, high-degree and third-degree AVB*.

During the lockdowns, there were statistically significant but weak correlations or no correlations between new COVID-19 cases and medical visits related to diseases with severe symptoms, such as severe AVB (r = −0.495, *p* < 0.001) and ventricular tachycardia (r = −0.055, *p* = 0.614). Negative correlations became prominent in diseases with mild symptoms, such as ventricular extrasystole (r = −0.816, *p* < 0.001) and sinus bradycardia (r = −0.877, *p* < 0.001). But these correlations disappeared during the post-lockdown and post-SARS-CoV-2 periods (Supplementary Table 9 and Supplementary Figure 8).

## Discussion

Based on a large volume of ECG data, we investigated the impact of the COVID-19 epidemic on the health-seeking behavior of patients with cardiac arrhythmias in Shanghai, China. The main findings of this study are as follows. First, the health-seeking behavior of patients with suspected cardiac arrhythmias, reflected by medical visits with ECG examinations, exhibited a decrease-rebound-fallback pattern during the period starting from the COVID-19 lockdowns. This decrease in visits during the lockdown period was largely attributed to reduced patient volume at community clinics, whereas visits to academic hospitals were less affected. Second, a negative correlation was found between the number of new COVID-19 cases or deaths and medical visits on the same day as well as during the following six weeks during the lockdowns; these correlations were most prominent within the seven days after the report of new COVID-19 cases or deaths. Third, the impact of COVID-19 on health-seeking behavior varied with the types of the arrhythmias, and the medical demand for arrhythmias with potentially severe symptoms was less affected by the epidemic.

### Decrease-Rebound-Fallback Pattern Following COVID-19 Lockdowns

During the initial phase of the COVID-19 outbreak, in the absence of vaccines or effective treatment protocols, self-isolation, as a standard quarantine measure, proved to be the most effective non-medical means to stop the spread of the virus. As a surging number of COVID-19 patients overwhelmed medical resources during the early stage of the epidemic, the Chinese government called for non-COVID-19 patients with mild symptoms to stay at home to reserve the capacity of medical institutions for patients with COVID-19 or for patients with severe diseases and symptoms. Medical institutions also provided online consultations with doctors to help patients identify urgent situations.

Similar to the findings of previous studies in China and other countries (10, 14–18) medical visits in Shanghai, an area outside the epidemic's epicenter, also decreased by approximately 40% during the Wuhan lockdown period. But the suppressed health-seeking demand from non-COVID-19 diseases was released after the lockdown period, forming a drastic post-lockdown surge. This fall-rebound pattern was also reported by a study from Israel in that noted a decrease in hospital admissions for myocardial infarction was observed during the early stage of the epidemic, as well as a rebounding increase upon the receding end of the first wave of the epidemic (11) Another study from Korea also showed that the number of outpatient visits in internal medicine decreased during the COVID-19 pandemic and tended to rebound during the second half of the year (19) Subsequently, medical visits gradually fell back to the baseline level of prior years, reflecting the normalization of health-seeking demand during the post-SARS-CoV-2 period in China.

New cases and deaths reported daily exerted a negative influence on the health-seeking behavior of patients over the following 6 weeks, and this trend was especially strong the first week after the report. It is also of note that this negative correlation between reported new cases or deaths with medical visits was detected based on new cases or deaths reported for the whole of China rather than just Shanghai locally (Supplementary Table 10 and Supplementary Figure 9), which reflects the impact of uniform policy on the behavior pattern of patients across China. As new cases per day dropped into single digits and as Wuhan lifted outbound travel restrictions, the correlation between COVID-19 cases and medical visits weakened or even disappeared, reflecting a shift of public focus and a return to normal daily life.

In addition, the general decrease in medical visits during the lockdowns was largely attributed to the decrease in visits to community clinics rather than to academic hospitals. Considering the hierarchical medical system in China, community clinics are mainly responsible for the long-term management of chronic diseases with mild symptoms, whereas patients with critical conditions and severe symptoms are usually treated at academic hospitals. This hierarchical separation of functions at different medical institutions was supported by our data regarding medical visits concerning cardiac arrhythmias during the pre-COVID-19 years (Supplementary Table 11). Such health-seeking preferences regarding academic hospitals and community clinics were amplified during the COVID-19 epidemic, suggesting that urgent conditions drove patients to admit themselves to academic hospitals regardless of the suggestions to isolate at home; medical visits by arrhythmic patients with mild symptoms or chronic conditions, however, largely decreased due to concerns over nosocomial infection by COVID-19.

### Behavioral Differences Exhibited Among Various Arrhythmias

Health-seeking behavior differed among various arrhythmias regarding the number of medical visits during and following the COVID-19 epidemic. Three patterns were detected. Pattern 1 was named the “stable-increase-stable” pattern and is represented by diseases such as severe AVB and atrial fibrillation (Supplementary Figure 6). The number of such medical visits remained stable during the lockdowns, followed by a post-lockdown increase and a return to baseline levels during the post-SARS-CoV-2 period. No prominent cosine-like curve in medical visits was observed. Patients with diseases conforming to Pattern 1 often manifested with severe symptoms and urgent conditions, and they tended to seek health services regardless of their concerns about nosocomial infection with COVID-19, thus reflecting a rigid medical demand. Pattern 2 was named the “stable-stable-stable” pattern and is represented by VT. This pattern corresponded to a relatively constant level of medical visits during and following the epidemic. As the ECG diagnostic system did not differentiate between non-sustained VT as short as three beats and sustained VT, which can cause hemodynamic disorders, it is possible that this “stable” level represents the mixed effects of situations of different clinical severities. Pattern 3 was named the “decrease-rebound-fallback” pattern, i.e., a cosine-like curve, and is represented by sinus bradycardia and the conditions in Supplementary Figure 7. In this pattern, patients with arrhythmias exhibiting mild symptoms tended to follow home isolation suggestions during the lockdowns. Medical demand, however, was not suppressed after the lockdown, as a rebound in medical visits occurred. As COVID-19 was further controlled in China, health-seeking behavior returned to the rational levels of prior years. Taken together, these health-seeking behaviors during and following the epidemic were not uniform for different diseases. A rigid medical demand for some diseases, such as severe AVB and VT, was revealed by unchanging or increased medical visits during the epidemic. Our results thus highlight the importance of medical institutions coping with non-COVID-19 -but nonetheless severe - diseases during the epidemic, as well as the importance of preparing for a surge in medical visits for various arrhythmias during the post-SARS-CoV-2 period.

### Implications

China's anti-COVID-19 measures included city-wide lockdowns, transportation freezes or controls in hard-hit areas, the timely release of COVID-19 information, the prevention of social gatherings and infections, thorough community screening, the quarantining of suspected individuals, the early admission and treatment of confirmed cases, extensive epidemiological investigations and a tremendous number of other efforts aimed at controlling the epidemic, such as the manufacture of sufficient medical products and vaccine research and development (20–25) As COVID-19 exerted negative effects on non-COVID diseases (7, 10) the early control of COVID-19 was also of great significance in improving the prognoses of patients with cardiac arrhythmias.

Considering that COVID-19 is still prevalent around the world and that China is now facing a new wave of Omicron variants recently, the results of our research may have important implications for China and other countries in planning the allocation of medical resources during these new epidemic and post-SARS-CoV-2 periods. Our results show that the number of medical visits will exhibit a post-SARS-CoV-2 surge that might last for nearly half a year. As an economically developed area in China, Shanghai properly handled the prior post-SARS-CoV-2 surge in medical demand. There might be an imbalance between medical supply and demand, however, during another post-SARS-CoV-2 period in less-developed regions and rural areas with fewer medical resources. Given the rapidly expanding vaccination process, the COVID-19 pandemic is expected to be taken under control around the world in the near future. Governments and medical institutions should pay great attention to preemptively coping with post-COVID surges in medical demand among patients with cardiac arrhythmias. In addition, this might also be true for patients with other diseases.

### Limitations

As the corresponding clinical, laboratory and imaging data were lacking, diagnoses made by ECG alone will result in a certain number of misdiagnoses and missed diagnoses, as well as conditions beyond arrhythmias. However, the diagnoses of cardiac arrhythmias highly relied on ECG. Second, a 30-second ECG is not able to discriminate between subtypes or between the severity of symptoms of specific arrhythmias, such as non-sustained and sustained VT, and atrial fibrillation with a fast or normal range of ventricular rate. Sinus bradycardia in this system follows the ECG criteria in which the sinus rate must be slower than 60 beats per minute. But according to new clinical guidelines, sinus bradycardia is now defined by a heart rate of <50 beats per minute (26) Also, ECG examination could be avoided to prevent contact-related infection after COVID-19, especially during the lockdown periods, which might be more prominent in community clinics and in patients with mild symptoms. These factors might bias the findings of our study.

## Conclusions

The number of medical visits related to cardiac arrhythmias exhibited a decrease-rebound-fallback pattern during the period starting from the COVID-19 lockdown in Shanghai. During this lockdown period, the severity of the epidemic, reflected by daily new cases or deaths, exerted a negative 6-week impact on the patients' behaviors in their seeking of medical services, and this impact was most prominent during the week following the daily report of new cases or deaths. Medical visits for arrhythmias with potentially severe symptoms, such as severe AVB and VT, were not negatively affected by the epidemic, reflecting the rigid medical demand of these patients.

## Data Availability Statement

The original contributions presented in the study are included in the article/Supplementary Material, further inquiries can be directed to the corresponding author/s.

## Ethics Statement

The studies involving human participants were reviewed and approved by Ethics Committee of Xin Hua Hospital Affiliated to Shanghai Jiao Tong University School of Medicine. Written informed consent for participation was not required for this study in accordance with the national legislation and the institutional requirements.

## Author Contributions

CL, MuC, and ML reviewed literature, analyzed the data, drafted, revised the manuscript, and designed or coded the figures and tables. HWa and XH collected the data, conducted a literature review, and interpreted the data. YZhu, BZ, and XQ developed and maintained the platform for the remote ECG diagnosis system. QW, JS, and MY critically revised the diagnosis of ECG for further analyses. MiC, XL, YZha, MS, and JH provided critical feedback on data sources. LL and PL provided guidance and support for statistical methods. HWu provided support for the development of the ECG platform. YG-L designed the study, acquired funding and managed the project. All authors had final responsibility for the decision to submit this paper for publication.

## Funding

This study was supported by Shanghai Hospital Development Center (SHDC2020CR2026B), National Natural Science Foundation of China (Nos. 82130009 and 81900288), Shanghai Municipal Science and Technology Commission (Nos. 201409005600 and 19411963500), and Hospital Funded Clinical Research, Xinhua Hospital Affiliated to Shanghai Jiao Tong University School of Medicine (No. 19XHCR05B).

## Conflict of Interest

XQ, BZ, and MC were employed by the company Shanghai Siwei Medical Co. Ltd.

The remaining authors declare that the research was conducted in the absence of any commercial or financial relationships that could be construed as a potential conflict of interest.

## Publisher's Note

All claims expressed in this article are solely those of the authors and do not necessarily represent those of their affiliated organizations, or those of the publisher, the editors and the reviewers. Any product that may be evaluated in this article, or claim that may be made by its manufacturer, is not guaranteed or endorsed by the publisher.
