# Supplementary material for: ECG Utilization Patterns of Patients With Arrhythmias During COVID-19 Epidemic and Post-SARS-CoV-2 Eras in Shanghai, China
Source: Front Cardiovasc Med. 2022 Apr 27;9:829679. doi: 10.3389/fcvm.2022.829679 (PMC9091729; doi:10.3389/fcvm.2022.829679)
Supplement: Supplementary file 1 [file Data_Sheet_1.PDF]

Original Investigations

ECG utilization patterns of patients with arrhythmias during COVID-19 epidemic and post-SARS-CoV-2 eras in Shanghai, China

Cheng Li, *MD*[1†]; Mu Chen, *MD*[1†]; Mohan Li, *BS*[1,2†]; Haicheng Wang, *MD*[1]; Xiangjun Qiu, *ME*[3]; Xiaoliang Hu, *PhD*[1]; Qunshan Wang, *MD*[1]; Jian Sun, *MD*[1]; Mei Yang, *MD*[1]; Yuling Zhu, *MBA*[4]; Peng Liao, *PhD*[5]; Baohong Zhou, *MBBS*[3]; Min Chen, *MBA*[3]; Xia Liu, *MD*[4]; Yuelin Zhao[4], Mingzhen Shen[4], Jinkang Huang[4]; Li Luo, *PhD*[5]; Hong Wu, *MD*[6]; and Yi-Gang Li, *MD*[1,3]

†These authors contributed equally.

Corresponding Author
Yi-Gang Li, *MD, FHRS*
Department of Cardiology
Xinhua Hospital, School of Medicine, Shanghai Jiao Tong University
1665 Kongjiang Road, Shanghai, China 200092
E-mail: liyigang@xinhuamed.com.cn

Supplementary materials

Supplementary Methods

Details of the artificial intelligence-assistant ECG diagnostic algorithm

All the ECG data were collected from ECG machines (NIHON KOHDEN ECG-1250P, MINDRAY BenenHeart R12, EDAN SE-1201 and so on) certified by China Food and Drug Administration (CFDA), which involved the ECG data of the patient in resting state for 30 seconds and were transmitted automatically after the collection. All ECG data, vector data in DICOM format, were encrypted and transmitted through the network and there was no data loss in the transmission process. After collection and transmission of raw data in the corresponding community hospitals or academic hospitals, the invalid data were eliminated.

Convolutional Neural Network (CNN) was used in ECG artificial intelligence (AI)-assistant diagnosis. The stability and accuracy of the AI-assistant ECG diagnostic algorithm have been validated by the ECG platform. The AI algorithm was implemented based on Tensorflow 1.13.1. The hardware environment of the training process was NVIDIA GeForce RTX 2080 Ti and the operating system was Ubuntu 18.04. Take the algorithm for atrial fibrillation (AF) diagnosis as an example. A total of 100,000 samples was collected as the training set, which contained 10,000 AF and 90,000 non-AF samples. Another 5,000 samples were used as the testing set, which involved 500 AF patients. There was no intersection between the testing and the training sets. In the training process, Adam (learning_rate = 0.001) was used as the optimator. The maximum number of iterations was 50, and the loss-function was categorical_crossentropy. When the loss-function could not be further reduced (accuracy was set to 1e-5) or the maximum number of iterations was reached, the training process would be stopped. During the process of training, validation_split was set to 0.2 so that Tensorflow randomly divided 20% of the data as the validation set. After the training, the parameters of CNN were fixed and the data in the testing set was input for prediction. Similar algorithms were adopted in other ECG diagnoses.

Procedures for obtaining the results of ECG diagnosis are as follows: firstly, the ECG was analyzed by AI and preliminary diagnosis was drawn, and then the diagnosis

would be reexamined by two clinicians. If the diagnosis of two clinicians consisted with each other and agreed with the diagnosis from AI, the diagnosis would be reserved. If two clinicians agreed with each other but disagreed with diagnosis from AI, the diagnosis results of clinicians would be adopted. If one of the clinicians' diagnosis and AI diagnosis result were consistent but different from another physician's, the diagnosis from AI would be adopted. If the two clinicians' diagnosis discorded with each other and both of them were different from AI diagnosis, the ECG would be transfered to another physician and the final diagnosis would came from the same two of the four diagnosis above, which was rare in our research.

Statistical analysis

The numbers of daily medical visits involving ECG examinations were regarded as continuous variables and the visit numbers did not follow the normal distribution so that the Mann-Whitney non-parametric test was used to compare the changes in the number of ECG visits between the different time periods or subgroups.

Supplementary Table1. Provincial constitution of ECG data in China between 2018 and 2021.

Provinces	2018*	2019*	2020*	2021*	Total*
**Guangdong**	0.19%	1.12%	1.27%	1.57%	0.91%
**Guizhou**	1.86%	2.88%	4.31%	3.85%	3.10%
**Hainan**	<0.01%	<0.01%	<0.01%	<0.01%	<0.01%
**Hubei**	1.37%	0.94%	0.72%	0.87%	0.99%
**Hunan**	<0.01%	<0.01%	0.01%	<0.01%	<0.01%
**Jiangsu**	2.88%	2.96%	6.75%	4.82%	4.32%
**Jiangxi**	0.05%	0.05%	0.05%	0.12%	0.05%
**Sichuan**	<0.01%	<0.01%	<0.01%	<0.01%	<0.01%
**Tibet**	0.09%	0.17%	0.24%	0.40%	0.18%
**Xinjiang**	5.49%	3.27%	1.87%	3.01%	3.46%
**Yunnan**	0.50%	0.91%	1.18%	1.85%	0.92%
**Zhejiang**	0.08%	0.31%	0.90%	1.38%	0.49%
**Shanghai**	87.50%	87.40%	82.70%	82.13%	85.56%

* Percentage of the number of ECG in each province of China.

Supplementary Table 2. District constitution of ECG in Shanghai between 2018 and 2021.

Districts of Shanghai	2018*	2019*	2020*	2021*	Total*
**Baoshan**	1.02%	1.09%	0.48%	0.57%	0.84%
**Changning**	6.98%	7.79%	8.94%	9.00%	7.98%
**Chongming**	12.60%	11.92%	10.27%	8.32%	11.42%
**Hongkou**	0.04%	<0.01%	0.12%	<0.01%	0.05%
**Huangpu**	1.49%	2.11%	2.50%	3.53%	2.11%
**Jiading**	7.97%	7.51%	0.08%	<0.01%	4.84%
**Jingan**	0.30%	0.39%	0.43%	0.05%	0.36%
**Jinshan**	15.53%	10.83%	14.62%	15.29%	13.75%
**Minhang**	1.78%	2.62%	2.47%	5.06%	2.43%
**Pudong**	21.11%	22.04%	26.95%	24.60%	23.49%
**Putuo**	<0.01%	0.06%	0.01%	0.01%	0.02%
**Qingpu**	3.79%	4.04%	3.93%	4.83%	3.96%
**Songjiang**	18.85%	19.56%	19.61%	18.09%	19.29%
**Xuhui**	7.37%	8.88%	8.36%	9.62%	8.28%
**Yangpu**	1.18%	1.18%	1.21%	1.03%	1.18%

* Percentage of the number of ECG in each district of Shanghai.

Supplementary Table 3. Medical visits of subgroups in Shanghai between 2018 and 2021 (monthly).

	Sex		Age			Tier of medical institutions		Total
	Males	Females	≤59y	60-79y	≥80y	Academic hospitals	Community clinics	
**Baseline (average of 2018 and 2019)**								
**January**	13439	17065	12079	12661	5765	9574	20930	30504
**February**	11190	13904	9180	10873	5042	7613	17481	25094
**March**	20374	24438	17248	20633	6932	10981	33831	44812
**April**	31028	37508	16865	41993	9678	11300	57236	68535
**May**	53514	64971	22025	80890	15570	13346	105139	118485
**June**	48663	57227	20598	71362	13930	11962	93929	105890
**July**	37936	43560	21534	48879	11083	12603	68893	81496
**August**	24538	26664	18103	25546	7553	11677	39525	51202
**September**	22483	25796	17669	23251	7359	10857	37423	48279
**October**	19881	22183	17479	17994	6592	12038	30027	42064
**November**	20612	23078	18452	18634	6604	13805	29885	43690
**December**	18013	21833	15745	17249	6852	13330	26516	39846
**Total**	321669	378226	206975	389963	102957	139083	560812	699895
**2020**								
**January**	14387	16830	12247	12738	6232	13493	17724	31217
**February**	7187	8975	5373	7416	3373	6478	9684	16162
**March**	11531	15485	9945	11973	5098	10959	16057	27016
**April**	16376	20775	15111	15667	6373	15551	21600	37151
**May**	22533	27188	17781	24547	7393	17755	31966	49721
**June**	33689	40909	19597	45282	9719	18521	56077	74598
**July**	43801	52898	23428	61436	11835	21715	74984	96699
**August**	39384	49277	22621	54549	11491	19800	68861	88661
**September**	41464	51765	22908	59002	11319	19886	73343	93229
**October**	36438	44437	21730	48582	10563	19788	61087	80875
**November**	34333	41091	22264	43087	10073	19180	56244	75424
**December**	30116	37498	21254	36665	9695	22038	45576	67614
**Total**	331239	407128	214259	420944	103164	205164	533203	738367
**2021**								
**January**	13010	17136	11364	14247	4535	10181	19965	30146
**February**	9620	12201	8428	9841	3552	7785	14036	21821
**March**	23042	25362	19010	22704	6690	16300	32104	48404

Supplementary Table 4. Medical visits during the lockdown (2020) or the same period of 2019 and 2021 in Shanghai (weekly).

	Sex		Age			Tier of medical institutions		Total
	Males	Females	≤59 y	60-79 y	≥80 y	Academic hospitals	Community clinics	
**Baseline (average of 2018 and 2019)**								
**Week 1 (Jan 23-Jan 29)**	2809	3591	2450	2697	1253	1965	4434	6399
**Week 2 (Jan 30-Feb 5)**	2543	3220	2048	2544	1171	1792	3971	5763
**Week 3 (Feb 6-Feb 12)**	2324	3060	1789	2420	1175	1655	3729	5383
**Week 4(Feb 13-Feb 19)**	2219	2862	1848	2136	1097	1692	3389	5081
**Week 5 (Feb 20-Feb 26)**	3559	4314	2993	3390	1491	2241	5632	7873
**Week 6 (Feb 27-Mar 5)**	4173	4874	3713	3794	1541	2568	6479	9047
**Week 7 (Mar 6-Mar 12)**	4240	5004	3819	3901	1524	2438	6806	9243
**Week 8 (Mar 13-Mar 19)**	4890	5774	4294	4752	1618	2549	8115	10664
**Week 9 (Mar 20-Mar 26)**	5109	6200	4173	5514	1622	2568	8741	11309
**Week 10 (Mar 27-Apr 2)**	4815	5989	3483	5628	1693	2356	8448	10804
**Week 11 (Apr 3-Apr 7)**	2156	2513	1748	2197	725	1452	3217	4669
**Total**	38834	47398	32354	38971	14907	23273	62959	86232
**2020**								
**Week 1 (Jan 23-Jan 29)**	1440	1445	1095	1152	638	1636	1249	2885
**Week 2(Jan 30-Feb 5)**	1820	2034	1346	1648	860	1671	2183	3854
**Week 3 (Feb 6-Feb 12)**	1537	1863	1174	1505	721	1469	1931	3400
**Week 4 (Feb 13-Feb 19)**	1698	2097	1229	1802	764	1447	2348	3795
**Week 5 (Feb 20-Feb 26)**	1942	2718	1517	2211	932	1783	2877	4660
**Week 6 (Feb 27-Mar 5)**	2122	3051	1627	2522	1024	2063	3110	5173
**Week 7 (Mar 6-Mar 12)**	2361	3228	2056	2448	1085	2239	3350	5589
**Week 8 (Mar 13-Mar 19)**	2825	3830	2357	2983	1315	2655	4000	6655
**Week 9 (Mar 20-Mar 26)**	3170	3848	2771	2978	1269	2840	4178	7018
**Week 10 (Mar 27-Apr 2)**	2673	3723	2507	2734	1155	2723	3673	6396
**Week 11 (Apr 3-Apr 7)**	1717	2104	1606	1520	695	1705	2116	3821
**Total**	23305	29941	19285	23503	10458	22231	31015	53246
**2021**								
**Week 1 (Jan 23-Jan 29)**	2677	3675	2491	2885	976	2191	4161	6352
**Week 2 (Jan 30-Feb 5)**	2610	3547	2333	2896	928	1887	4270	6157
**Week 3 (Feb 6-Feb 12)**	1891	2213	1670	1761	673	1660	2444	4104
**Week 4 (Feb 13-Feb 19)**	1614	2017	1372	1577	682	1664	1967	3631
**Week 5 (Feb 20-Feb 26)**	3433	4400	2994	3562	1277	2530	5303	7833
**Week 6 (Feb 27-Mar 5)**	3748	5022	3270	4158	1342	2782	5988	8770
**Week 7 (Mar 6-Mar 12)**	4657	4950	4436	3951	1220	3534	6073	9607
**Week 8 (Mar 13-Mar 19)**	5256	5419	4545	4750	1380	3937	6738	10675
**Week 9 (Mar 20-Mar 26)**	5942	6270	4475	6229	1508	3983	8229	12212
**Week 10 (Mar 27-Apr 2)**	5890	6380	4098	6243	1929	3809	8461	12270
**Week 11 (Apr 3-Apr 7)**	2695	2872	2171	2648	748	2180	3387	5567
**Total**	40413	46765	33855	40660	12663	30157	57021	87178

Supplementary table 5. Correlation between the number of new COVID-19 cases or deaths and medical visits during and after the lockdown in Shanghai.

	Lockdown *				After Lockdown †			
	New COVID-19 cases		New deaths		New COVID-19 cases		New deaths	
	r ‡	p value	r ‡	p value	r ‡	p value	r ‡	p value
**During the following 3 days (total)**	-0.765	<0.001	-0.688	<0.001	-0.030	0.578	-0.159	0.002
**During the following 7 days (total)**	-0.873	<0.001	-0.724	<0.001	-0.052	0.331	-0.164	0.002
**During the following 14 days (total)**	-0.804	<0.001	-0.603	<0.001	-0.097	0.070	-0.169	0.002
**During the following 21 days (total)**	-0.693	<0.001	-0.491	<0.001	-0.138	0.011	-0.185	0.001
**During the following 3 days (males)**	-0.747	<0.001	-0.702	<0.001	-0.037	0.485	-0.161	0.002
**During the following 7 days (males)**	-0.876	<0.001	-0.761	<0.001	-0.055	0.300	-0.167	0.002
**During the following 14 days (males)**	-0.842	<0.001	-0.675	<0.001	-0.102	0.055	-0.174	0.001
**During the following 21 days (males)**	-0.743	<0.001	-0.564	<0.001	-0.142	0.008	-0.187	<0.001
**During the following 3 days (females)**	-0.776	<0.001	-0.665	<0.001	-0.024	0.656	-0.155	0.003
**During the following 7 days (females)**	-0.861	<0.001	-0.697	<0.001	-0.042	0.426	-0.158	0.003
**During the following 14 days (females)**	-0.767	<0.001	-0.551	<0.001	-0.091	0.088	-0.164	0.002
**During the following 21 days (females)**	-0.650	<0.001	-0.435	<0.001	-0.132	0.015	-0.181	0.001
**During the following 3 days (academic hospitals)**	-0.803	<0.001	-0.800	<0.001	-0.114	0.030	-0.107	0.041
**During the following 7 days (academic hospitals)**	-0.882	<0.001	-0.830	<0.001	-0.118	0.026	-0.126	0.017
**During the following 14 days (academic hospitals)**	-0.856	<0.001	-0.739	<0.001	-0.137	0.010	-0.124	0.020
**During the following 21 days (academic hospitals)**	-0.772	<0.001	-0.612	<0.001	-0.160	0.003	-0.147	0.006
**During the following 3 days (community clinics)**	-0.734	<0.001	-0.612	<0.001	-0.017	0.753	-0.170	0.001
**During the following 7 days (community clinics)**	-0.856	<0.001	-0.662	<0.001	-0.038	0.478	-0.184	<0.001
**During the following 14 days (community clinics)**	-0.755	<0.001	-0.508	<0.001	-0.090	0.094	-0.184	0.001
**During the following 21 days (community clinics)**	-0.632	<0.001	-0.407	<0.001	-0.135	0.012	-0.200	<0.001
**During the following 3 days (<60 y)**	-0.806	<0.001	-0.766	<0.001	-0.098	0.064	-0.139	0.008
**During the following 7 days (<60 y)**	-0.867	<0.001	-0.773	<0.001	-0.076	0.154	-0.151	0.004
**During the following 14 days (<60 y)**	-0.807	<0.001	-0.668	<0.001	-0.106	0.048	-0.154	0.004
**During the following 21 days (<60 y)**	-0.737	<0.001	-0.551	<0.001	-0.109	0.043	-0.174	0.001
**During the following 3 days (60-79 y)**	-0.709	<0.001	-0.596	<0.001	-0.019	0.713	-0.168	0.001
**During the following 7 days (60-79 y)**	-0.862	<0.001	-0.673	<0.001	-0.049	0.352	-0.178	0.001
**During the following 14 days (60-79 y)**	-0.757	<0.001	-0.504	<0.001	-0.103	0.053	-0.181	0.001
**During the following 21 days (60-79 y)**	-0.627	<0.001	-0.398	<0.001	-0.149	0.006	-0.194	<0.001
**During the following 3 days (>79 y)**	-0.729	<0.001	-0.658	<0.001	-0.013	0.810	-0.122	0.020
**During the following 7 days (>79 y)**	-0.886	<0.001	-0.725	<0.001	-0.010	0.844	-0.131	0.013
**During the following 14 days (>79 y)**	-0.844	<0.001	-0.638	<0.001	-0.049	0.364	-0.142	0.008
**During the following 21 days (>79 y)**	-0.733	<0.001	-0.549	<0.001	-0.078	0.151	-0.161	0.003

* Between January 23 and April 7, 2020

† Between April 8 and April 7, 2021

‡ Spearman's rank correlation coefficient. The comparisons were obtained by Mann-Whitney test.

Supplementary Table 6. Correlation between the number of new COVID-19 discharged or existing confirmed cases and medical visits during and after the lockdown in Shanghai.

	Lockdown *				After Lockdown †			
	New discharged cases		Existing confirmed cases		New discharged cases		Existing confirmed cases	
	r ‡	p value	r ‡	p value	r ‡	p value	r ‡	p value
**During the following 3 days (total)**	-0.179	0.095	-0.523	<0.001	-0.239	<0.001	-0.262	<0.001
**During the following 7 days (total)**	-0.085	0.434	-0.503	<0.001	-0.265	<0.001	-0.309	<0.001
**During the following 14 days (total)**	0.103	0.339	-0.348	0.001	-0.291	<0.001	-0.348	<0.001
**During the following 3 days (males)**	-0.251	0.018	-0.561	<0.001	-0.255	<0.001	-0.275	<0.001
**During the following 7 days (males)**	-0.155	0.150	-0.560	<0.001	-0.278	<0.001	-0.313	<0.001
**During the following 14 days (males)**	0.017	0.872	-0.434	<0.001	-0.302	<0.001	-0.351	<0.001
**During the following 3 days (females)**	-0.116	0.283	-0.485	<0.001	-0.222	<0.001	-0.251	<0.001
**During the following 7 days (females)**	-0.041	0.706	-0.464	<0.001	-0.251	<0.001	-0.301	<0.001
**During the following 14 days (females)**	0.160	0.136	-0.288	0.007	-0.282	<0.001	-0.340	<0.001
**During the following 3 days (academic hospitals)**	-0.329	0.002	-0.681	<0.001	-0.255	<0.001	-0.278	<0.001
**During the following 7 days (academic hospitals)**	-0.242	0.023	-0.658	<0.001	-0.293	<0.001	-0.298	<0.001
**During the following 14 days (academic hospitals)**	-0.055	0.608	-0.520	<0.001	-0.323	<0.001	-0.308	<0.001
**During the following 3 days (community clinics)**	-0.097	0.368	-0.435	<0.001	-0.237	<0.001	-0.261	<0.001
**During the following 7 days (community clinics)**	-0.013	0.907	-0.426	<0.001	-0.269	<0.001	-0.315	<0.001
**During the following 14 days (community clinics)**	0.199	0.063	-0.243	0.023	-0.293	<0.001	-0.362	<0.001
**During the following 3 days (< 60 y)**	-0.267	0.012	-0.617	<0.001	-0.222	<0.001	-0.291	<0.001
**During the following 7 days (< 60 y)**	-0.152	0.157	-0.570	<0.001	-0.215	<0.001	-0.288	<0.001
**During the following 14 days (< 60 y)**	0.034	0.754	-0.424	<0.001	-0.224	<0.001	-0.304	<0.001
**During the following 3 days (60-79 y)**	-0.085	0.433	-0.422	<0.001	-0.249	<0.001	-0.262	<0.001
**During the following 7 days (60-79 y)**	-0.014	0.897	-0.434	<0.001	-0.286	<0.001	-0.319	<0.001
**During the following 14 days (60-79 y)**	0.204	0.056	-0.240	0.025	-0.309	<0.001	-0.360	<0.001
**During the following 3 days (> 79 y)**	-0.197	0.066	-0.511	<0.001	-0.189	<0.001	-0.223	<0.001
**During the following 7 days (> 79 y)**	-0.115	0.286	-0.517	<0.001	-0.199	<0.001	-0.246	<0.001
**During the following 14 days (> 79 y)**	0.051	0.634	-0.395	<0.001	-0.241	<0.001	-0.280	<0.001

* Between January 23 and April 7, 2020

† Between April 8 and April 7, 2021

‡ Spearman's rank correlation coefficient.

Supplementary Table 7. Correlation between the number of new COVID-19 cases or deaths and medical visits in Shanghai before or after the Spring Festival, 2020.

	Before Spring Festival, 2020 *				After Spring Festival, 2020 †			
	New cases		New deaths		New cases		New deaths	
	r ‡	p value	r ‡	p value	r ‡	p value	r ‡	p value
**During the following 3 days (total)**	-0.701	0.011	-0.772	0.003	-0.719	<0.001	-0.665	<0.001
**During the following 7 days (total)**	-0.947	<0.001	-0.880	<0.001	-0.865	<0.001	-0.846	<0.001
**During the following 14 days (total)**	-0.819	0.001	-0.747	0.005	-0.885	<0.001	-0.876	<0.001
**During the following 3 days (males)**	-0.715	0.009	-0.735	0.006	-0.700	<0.001	-0.671	<0.001
**During the following 7 days (males)**	-0.947	<0.001	-0.880	<0.001	-0.855	<0.001	-0.847	<0.001
**During the following 14 days (males)**	-0.826	0.001	-0.728	0.007	-0.886	<0.001	-0.878	<0.001
**During the following 3 days (females)**	-0.701	0.011	-0.772	0.003	-0.734	<0.001	-0.647	<0.001
**During the following 7 days (females)**	-0.947	<0.001	-0.880	<0.001	-0.866	<0.001	-0.843	<0.001
**During the following 14 days (females)**	-0.819	0.001	-0.747	0.005	-0.883	<0.001	-0.876	<0.001
**During the following 3 days (academic hospitals)**	-0.687	0.014	-0.673	0.017	-0.776	<0.001	-0.779	<0.001
**During the following 7 days (academic hospitals)**	-0.948	<0.001	-0.881	<0.001	-0.846	<0.001	-0.899	<0.001
**During the following 14 days (academic hospitals)**	-0.819	0.001	-0.747	0.005	-0.852	<0.001	-0.908	<0.001
**During the following 3 days (community clinics)**	-0.755	0.005	-0.806	0.002	-0.670	<0.001	-0.575	<0.001
**During the following 7 days (community clinics)**	-0.947	<0.001	-0.880	<0.001	-0.867	<0.001	-0.804	<0.001
**During the following 14 days (community clinics)**	-0.826	0.001	-0.728	0.007	-0.892	<0.001	-0.852	<0.001
**During the following 3 days (<60 y)**	-0.804	0.002	-0.791	0.002	-0.787	<0.001	-0.785	<0.001
**During the following 7 days (<60 y)**	-0.915	<0.001	-0.846	0.001	-0.834	<0.001	-0.892	<0.001
**During the following 14 days (<60 y)**	-0.819	0.001	-0.747	0.005	-0.843	<0.001	-0.910	<0.001
**During the following 3 days (60-79 y)**	-0.609	0.036	-0.687	0.014	-0.629	<0.001	-0.537	<0.001
**During the following 7 days (60-79 y)**	-0.961	<0.001	-0.880	<0.001	-0.870	<0.001	-0.802	<0.001
**During the following 14 days (60-79 y)**	-0.819	0.001	-0.747	0.005	-0.892	<0.001	-0.842	<0.001
**During the following 3 days (>79 y)**	-0.680	0.015	-0.706	0.010	-0.677	<0.001	-0.606	<0.001
**During the following 7 days (>79 y)**	-0.947	<0.001	-0.880	<0.001	-0.897	<0.001	-0.807	<0.001
**During the following 14 days (>79 y)**	-0.819	0.001	-0.747	0.005	-0.895	<0.001	-0.836	<0.001

* Between January 11 and January 22, 2020

† Between February 3 and April 7, 2020

‡ Spearman's rank correlation coefficient.

Supplementary Table 8. Monthly medical visits of various cardiac arrhythmias in Shanghai (2018-2021).

	Jan	Feb	Mar	Apr	May	Jun	Jul	Aug	Sep	Oct	Nov	Dec	Overall
**Baseline (average of 2018 and 2019)**													
**Normal ECG**	12539	10262	20454	31006	53857	47861	37025	22820	20825	17896	18914	16656	310113
**Sinus bradycardia**	1564	1184	2868	6233	12261	11404	7788	5227	5031	4189	3814	2704	64264
**Sinus tachycardia**	2917	2439	2614	2532	3027	2523	2435	1784	1879	1786	2053	2514	28500
**Atrial extrasystole**	2307	2051	2926	4452	7418	6631	4913	2894	2793	2552	2629	2654	44217
**Atrial tachycardia**	259	227	265	344	509	435	340	248	234	236	234	245	3574
**Atrial flutter**	107	89	116	120	186	159	136	110	121	120	126	140	1527
**Atrial fibrillation**	1299	1072	1427	1867	2770	2605	2141	1490	1464	1395	1391	1469	20387
**Ventricular extrasystole**	1476	1288	1859	2592	3999	3609	2785	1830	1828	1717	1818	1827	26625
**Ventricular tachycardia**	10	9	11	11	16	15	16	7	12	12	11	14	141
**Paroxysmal SVT**	75	55	84	115	150	143	121	79	87	85	92	86	1168
**First-degree AVB**	1025	875	1458	2352	4120	3696	2920	1971	1971	1760	1639	1513	25299
**Severe AVB**	18	17	28	28	43	40	34	33	25	23	28	33	348
**RBBB**	1596	1368	2188	3438	6201	5791	4294	2719	2628	2240	2204	2219	36882
**LBBB**	139	127	206	326	544	515	382	243	230	198	211	191	3309
**Left anterior fascicular block**	340	270	418	712	1163	1044	766	418	435	434	425	440	6863
**2020**													
**Normal ECG**	12062	5762	10599	15575	21360	32318	43306	39308	40937	34922	33010	28770	317929
**Sinus bradycardia**	1793	815	1742	2876	5103	7781	10172	8757	9931	8263	7484	4856	69573
**Sinus tachycardia**	2760	1561	1944	2036	1996	2405	2740	2621	2686	2658	2571	3484	29462
**Atrial extrasystole**	2466	1331	1976	2512	3018	4591	5702	5242	5517	4682	4434	4254	45725
**Atrial tachycardia**	307	155	213	226	270	351	447	403	406	361	346	355	3840
**Atrial flutter**	147	91	137	165	178	205	265	180	229	198	208	201	2204
**Atrial fibrillation**	1398	820	1201	1458	1593	2011	2424	2264	2278	2169	2132	2312	22060
**Ventricular extrasystole**	1543	896	1369	1773	1936	2790	3421	3267	3392	2933	2890	2772	28982
**Ventricular tachycardia**	17	9	12	10	17	21	13	9	27	26	18	27	206
**Paroxysmal SVT**	72	52	86	85	111	131	154	148	163	157	105	127	1391
**First-degree AVB**	1308	754	1206	1529	2145	3007	3637	3404	3663	3248	3085	2596	29582
**Severe AVB**	30	18	30	37	49	49	47	44	60	40	43	48	495
**RBBB**	1795	1007	1547	2051	2678	4015	5119	4773	4930	4643	4311	3836	40705
**LBBB**	181	97	166	196	282	382	474	414	461	406	389	362	3810
**Left anterior fascicular block**	303	180	249	330	413	644	825	706	796	642	696	668	6452
**2021**													
**Normal ECG**	12746	9082	21253	/	/	/	/	/	/	/	/	/	43081
**Sinus bradycardia**	1851	1342	3348	/	/	/	/	/	/	/	/	/	9729
**Sinus tachycardia**	2187	1498	2532	/	/	/	/	/	/	/	/	/	7779
**Atrial extrasystole**	1950	1543	2953	/	/	/	/	/	/	/	/	/	7893
**Atrial tachycardia**	187	165	243	/	/	/	/	/	/	/	/	/	728
**Atrial flutter**	111	73	125	/	/	/	/	/	/	/	/	/	389
**Atrial fibrillation**	967	833	1374	/	/	/	/	/	/	/	/	/	3926
**Ventricular extrasystole**	1387	1025	2008	/	/	/	/	/	/	/	/	/	5441
**Ventricular tachycardia**	10	11	9	/	/	/	/	/	/	/	/	/	38

**Paroxysmal SVT**	73	51	97	/	/	/	/	/	/	/	/	/	275
**First-degree AVB**	1090	875	1845	/	/	/	/	/	/	/	/	/	4643
**Severe AVB**	27	26	24	/	/	/	/	/	/	/	/	/	93
**RBBB**	1599	1195	2556	/	/	/	/	/	/	/	/	/	6578
**LBBB**	141	100	254	/	/	/	/	/	/	/	/	/	596
**Left anterior fascicular block**	272	223	437	/	/	/	/	/	/	/	/	/	1139

The severe AVB includes second-degree type 2, high-degree and third-degree AVB.

SVT, supraventricular tachycardia; AVB, atrioventricular block; RBBB, right bundle branch block; LBBB, left bundle branch block.

Supplementary Table 9. Correlation between the number of new COVID-19 cases, new deaths, new discharged or existing confirmed cases and the number of medical visits of various cardiac arrhythmias during and after lockdown in Shanghai.

	Lockdown *								After lockdown †							
	New cases		New deaths		New discharged		Existing cases		New cases		New deaths		New discharged		Existing cases	
	r ‡	p	r ‡	p	r ‡	p	r ‡	p	r ‡	p	r ‡	p	r ‡	p	r ‡	p
**Medical visits of the following 3 days**																
**Normal ECG**	-0.802	<0.001	-0.712	<0.001	-0.172	0.108	-0.538	<0.001	-0.034	0.519	-0.159	0.002	-0.240	<0.001	-0.261	<0.001
**Sinus bradycardia**	-0.776	<0.001	-0.742	<0.001	-0.182	0.090	-0.553	<0.001	-0.072	0.172	-0.151	0.004	-0.257	<0.001	-0.302	<0.001
**Sinus tachycardia**	-0.448	<0.001	-0.423	<0.001	-0.352	0.001	-0.461	<0.001	0.084	0.111	-0.077	0.142	-0.180	0.001	-0.160	0.002
**Atrial extrasystole**	-0.685	<0.001	-0.629	<0.001	-0.172	0.109	-0.488	<0.001	-0.014	0.796	-0.142	0.007	-0.223	<0.001	-0.242	<0.001
**Atrial tachycardia**	-0.475	<0.001	-0.437	<0.001	-0.190	0.076	-0.365	<0.001	-0.043	0.412	-0.141	0.007	-0.204	<0.001	-0.221	<0.001
**Atrial flutter**	-0.500	<0.001	-0.403	<0.001	-0.067	0.534	-0.272	0.010	-0.067	0.203	-0.024	0.650	-0.237	<0.001	-0.176	0.001
**Atrial fibrillation**	-0.669	<0.001	-0.637	<0.001	-0.307	0.004	-0.548	<0.001	-0.004	0.938	-0.116	0.028	-0.193	<0.001	-0.200	<0.001
**Ventricular extrasystole**	-0.719	<0.001	-0.673	<0.001	-0.218	0.041	-0.527	<0.001	-0.019	0.716	-0.144	0.006	-0.209	<0.001	-0.233	<0.001
**Ventricular tachycardia**	-0.008	0.942	-0.154	0.152	-0.151	0.160	-0.128	0.236	-0.136	0.009	-0.143	0.006	-0.226	<0.001	-0.195	<0.001
**Paroxysmal SVT**	-0.286	0.007	-0.230	0.031	0.121	0.263	-0.058	0.595	0.013	0.804	-0.090	0.087	-0.233	<0.001	-0.294	<0.001
**First-degree AVB**	-0.694	<0.001	-0.612	<0.001	-0.078	0.468	-0.431	<0.001	-0.066	0.210	-0.152	0.004	-0.217	<0.001	-0.280	<0.001
**Severe AVB**	-0.335	0.001	-0.253	0.017	-0.019	0.860	-0.159	0.138	-0.008	0.873	0.022	0.670	-0.020	0.706	-0.111	0.035
**RBBB**	-0.730	<0.001	-0.676	<0.001	-0.197	0.066	-0.512	<0.001	-0.002	0.963	-0.156	0.003	-0.214	<0.001	-0.237	<0.001
**LBBB**	-0.614	<0.001	-0.523	<0.001	-0.177	0.098	-0.425	<0.001	-0.061	0.244	-0.148	0.005	-0.294	<0.001	-0.304	<0.001
**Left anterior fascicular block**	-0.514	<0.001	-0.423	<0.001	-0.202	0.060	-0.352	0.001	-0.022	0.679	-0.160	0.002	-0.241	<0.001	-0.246	<0.001
**Medical visits of the following 7 days**																
**Normal ECG**	-0.862	<0.001	-0.719	<0.001	-0.074	0.491	-0.495	<0.001	-0.056	0.290	-0.168	0.001	-0.268	<0.001	-0.306	<0.001
**Sinus bradycardia**	-0.816	<0.001	-0.695	<0.001	-0.057	0.598	-0.475	<0.001	-0.113	0.033	-0.153	0.004	-0.291	<0.001	-0.352	<0.001
**Sinus tachycardia**	-0.634	<0.001	-0.590	<0.001	-0.354	0.001	-0.574	<0.001	0.072	0.174	-0.127	0.016	-0.221	<0.001	-0.213	<0.001
**Atrial extrasystole**	-0.852	<0.001	-0.736	<0.001	-0.131	0.222	-0.534	<0.001	-0.033	0.539	-0.145	0.006	-0.246	<0.001	-0.282	<0.001
**Atrial tachycardia**	-0.580	<0.001	-0.516	<0.001	-0.056	0.603	-0.329	0.002	-0.052	0.328	-0.134	0.011	-0.246	<0.001	-0.250	<0.001
**Atrial flutter**	-0.679	<0.001	-0.490	<0.001	0.043	0.694	-0.305	0.004	-0.077	0.147	-0.031	0.558	-0.291	<0.001	-0.233	<0.001
**Atrial fibrillation**	-0.864	<0.001	-0.757	<0.001	-0.265	0.013	-0.604	<0.001	0.004	0.935	-0.123	0.019	-0.216	<0.001	-0.224	<0.001
**Ventricular extrasystole**	-0.877	<0.001	-0.740	<0.001	-0.139	0.198	-0.542	<0.001	-0.028	0.599	-0.155	0.003	-0.230	<0.001	-0.272	<0.001
**Ventricular tachycardia**	0.055	0.614	-0.132	0.219	-0.119	0.270	-0.115	0.287	-0.193	<0.001	-0.112	0.034	-0.272	<0.001	-0.258	<0.001
**Paroxysmal SVT**	-0.497	<0.001	-0.307	0.004	0.245	0.021	-0.068	0.528	-0.032	0.545	-0.099	0.062	-0.257	<0.001	-0.372	<0.001
**First-degree AVB**	-0.828	<0.001	-0.660	<0.001	-0.017	0.877	-0.428	<0.001	-0.077	0.147	-0.165	0.002	-0.240	<0.001	-0.318	<0.001
**Severe AVB**	-0.495	<0.001	-0.333	0.002	0.111	0.303	-0.202	0.059	-0.058	0.273	0.025	0.637	-0.053	0.319	-0.175	0.001
**RBBB**	-0.873	<0.001	-0.718	<0.001	-0.097	0.367	-0.499	<0.001	-0.014	0.788	-0.158	0.003	-0.231	<0.001	-0.278	<0.001
**LBBB**	-0.808	<0.001	-0.609	<0.001	-0.043	0.691	-0.429	<0.001	-0.063	0.232	-0.153	0.004	-0.326	<0.001	-0.339	<0.001
**Left anterior fascicular block**	-0.731	<0.001	-0.571	<0.001	-0.157	0.145	-0.441	<0.001	-0.045	0.393	-0.183	<0.001	-0.258	<0.001	-0.302	<0.001
**Medical visits of the following 14 days**																
**Normal ECG**	-0.780	<0.001	-0.583	<0.001	0.136	0.205	-0.323	0.002	-0.101	0.058	-0.172	0.001	-0.292	<0.001	-0.342	<0.001

**Sinus bradycardia**	-0.764	<0.001	-0.556	<0.001	0.157	0.144	-0.294	0.005	-0.153	0.004	-0.150	0.005	-0.304	<0.001	-0.370	<0.001
**Sinus tachycardia**	-0.734	<0.001	-0.717	<0.001	-0.285	0.007	-0.647	<0.001	0.043	0.421	-0.178	0.001	-0.250	<0.001	-0.241	<0.001
**Atrial extrasystole**	-0.853	<0.001	-0.651	<0.001	0.027	0.802	-0.413	<0.001	-0.084	0.115	-0.152	0.004	-0.286	<0.001	-0.333	<0.001
**Atrial tachycardia**	-0.793	<0.001	-0.616	<0.001	-0.003	0.975	-0.400	<0.001	-0.089	0.094	-0.148	0.005	-0.282	<0.001	-0.291	<0.001
**Atrial flutter**	-0.611	<0.001	-0.398	<0.001	0.227	0.033	-0.185	0.084	-0.106	0.048	-0.042	0.430	-0.345	<0.001	-0.296	<0.001
**Atrial fibrillation**	-0.887	<0.001	-0.730	<0.001	-0.070	0.517	-0.523	<0.001	-0.035	0.513	-0.129	0.015	-0.239	<0.001	-0.254	<0.001
**Ventricular extrasystole**	-0.807	<0.001	-0.627	<0.001	0.064	0.556	-0.381	<0.001	-0.064	0.230	-0.158	0.003	-0.257	<0.001	-0.313	<0.001
**Ventricular tachycardia**	-0.081	0.454	-0.070	0.519	-0.081	0.455	-0.063	0.562	-0.218	<0.001	-0.037	0.491	-0.264	<0.001	-0.334	<0.001
**Paroxysmal SVT**	-0.437	<0.001	-0.182	0.091	0.415	<0.001	0.066	0.542	-0.118	0.027	-0.144	0.007	-0.289	<0.001	-0.426	<0.001
**First-degree AVB**	-0.768	<0.001	-0.511	<0.001	0.199	0.064	-0.244	0.022	-0.112	0.037	-0.168	0.002	-0.269	<0.001	-0.347	<0.001
**Severe AVB**	-0.553	<0.001	-0.322	0.002	0.304	0.004	-0.112	0.297	-0.164	0.002	<0.001	0.998	-0.107	0.046	-0.259	<0.001
**RBBB**	-0.809	<0.001	-0.594	<0.001	0.104	0.337	-0.340	0.001	-0.056	0.298	-0.164	0.002	-0.261	<0.001	-0.314	<0.001
**LBBB**	-0.816	<0.001	-0.560	<0.001	0.148	0.168	-0.327	0.002	-0.110	0.040	-0.160	0.003	-0.351	<0.001	-0.383	<0.001
**Left anterior fascicular block**	-0.735	<0.001	-0.589	<0.001	0.033	0.761	-0.399	<0.001	-0.111	0.037	-0.192	<0.001	-0.278	<0.001	-0.341	<0.001

* Between January 23 and April 7, 2020

† Between April 8 and April 7, 2021

‡ Spearman's rank correlation coefficient.

The severe AVB includes second-degree type 2, high-degree and third-degree AVB.

SVT, supraventricular tachycardia; AVB, atrioventricular block; RBBB, right bundle branch block; LBBB, left bundle branch block.

Supplementary Table 10. Correlation between the number of new COVID-19 cases, new deaths, new discharged cases or existing confirmed cases in Shanghai and the total number of medical visits in Shanghai during and after the lockdown.

	Lockdown *								After lockdown †						
	New cases		New deaths		New discharged		Existing cases		New cases		New deaths §	New discharged		Existing cases	
	r ‡	p	r ‡	p	r ‡	p	r ‡	p	r ‡	p	/§	r ‡	p	r ‡	p
**During the following 3 days**	-0.225	0.045	0.104	0.357	-0.230	0.040	-0.336	0.002	-0.067	0.203	/§	-0.192	<0.001	-0.327	<0.001
**During the following 7 days**	-0.232	0.038	0.089	0.432	-0.099	0.384	-0.273	0.014	-0.049	0.357	/§	-0.234	<0.001	-0.362	<0.001
**During the following 14 days**	-0.274	0.014	0.091	0.422	0.018	0.878	-0.169	0.134	-0.080	0.134	/§	-0.258	<0.001	-0.431	<0.001

* Between January 23 and April 7, 2020

† Between April 8 and April 7, 2021

‡ Spearman's rank correlation coefficient.

§ After Apr. 7, 2020, the number of new deaths related to COVID-19 in Shanghai is zero.

Supplementary Table 11. Chi-square analyses of medical visits with various cardiac arrhythmias between academic hospitals and community clinics in different time periods.

	Cardiac arrhythmias	Community clinics, n(%)	Academic hospitals, n(%)	p value
**ECG events with more prominent symptoms**				
**Total (Jan 1, 2018 -Apr 7, 2021)**	Sinus tachycardia	57039(3.30)	36114(6.93)	<0.0001
	Atrial tachycardia	8986(0.52)	2654(0.51)	0.34
	Atrial flutter	4074(0.24)	1524(0.29)	<0.0001
	Atrial fibrillation	51298(2.97)	15002(2.88)	0.00073
	Ventricular tachycardia	282(0.02)	237(0.05)	<0.0001
	Paroxysmal SVT	2675(0.15)	1289(0.25)	<0.0001
	Severe AVB	913(0.05)	358(0.07)	<0.0001
**Baseline (Jan 23, 2018 -Apr 7, 2018 and Jan 23, 2019 -Apr 7, 2019)**	Sinus tachycardia	7623(6.05)	4987(10.71)	<0.0001
	Atrial tachycardia	926(0.74)	303(0.65)	0.064
	Atrial flutter	363(0.29)	155(0.33)	0.13
	Atrial fibrillation	4663(3.70)	1608(3.45)	0.014
	Ventricular tachycardia	35(0.03)	10(0.02)	0.47
	Paroxysmal SVT	224(0.18)	117(0.25)	0.0023
	Severe AVB	79(0.06)	29(0.06)	0.97
**Baseline (Apr 8, 2018-Dec 31,2018 and Apr 8, 2019-Dec 31,2019)**	Sinus tachycardia	25908(2.68)	14344(6.59)	<0.0001
	Atrial tachycardia	4547(0.47)	1011(0.46)	0.70
	Atrial flutter	1824(0.19)	562(0.26)	<0.0001
	Atrial fibrillation	26880(2.78)	5787(2.66)	0.0015
	Ventricular tachycardia	132(0.01)	88(0.04)	<0.0001
	Paroxysmal SVT	1379(0.14)	500(0.23)	<0.0001
	Severe AVB	442(0.05)	121(0.06)	0.057
**Lockdown (Jan 23, 2020 -Apr 7, 2020)**	Sinus tachycardia	2219(7.15)	2198(9.89)	<0.0001
	Atrial tachycardia	296(0.95)	153(0.69)	0.00093
	Atrial flutter	173(0.56)	98(0.44)	0.061
	Atrial fibrillation	1561(5.03)	974(4.38)	0.00050
	Ventricular tachycardia	8(0.03)	15(0.07)	0.022
	Paroxysmal SVT	74(0.24)	89(0.40)	0.00086
	Severe AVB	36(0.12)	22(0.10)	0.56
**Post-lockdown era (Apr 8, 2020 -Dec 31, 2020)**	Sinus tachycardia	14171(2.91)	8650(5.04)	<0.0001
	Atrial tachycardia	2342(0.48)	788(0.46)	0.25
	Atrial flutter	1293(0.27)	509(0.30)	0.035
	Atrial fibrillation	13733(2.82)	4641(2.71)	0.011
	Ventricular tachycardia	76(0.02)	92(0.05)	<0.0001
	Paroxysmal SVT	766(0.16)	401(0.23)	<0.0001
	Severe AVB	279(0.06)	134(0.08)	0.0032
**Post-SARS-CoV-2 era (Jan 23-Apr 7, 2021)**	Sinus tachycardia	2767(4.85)	2234(7.41)	<0.0001
	Atrial tachycardia	365(0.64)	142(0.47)	0.0018
	Atrial flutter	191(0.33)	69(0.23)	0.0063
	Atrial fibrillation	1927(3.38)	806(2.67)	<0.0001
	Ventricular tachycardia	14(0.02)	12(0.04)	0.22
	Paroxysmal SVT	107(0.19)	76(0.25)	0.048
	Severe AVB	39(0.07)	23(0.08)	0.68
**ECG events with less prominent symptoms**				
**Total (Jan 1, 2018 -Apr 7, 2021)**	Normal ECG	737517(42.70)	247951(47.59)	<0.0001
	Sinus bradycardia	159309(9.22)	46173(8.86)	<0.0001
	Atrial extrasystole	117233(6.79)	23970(4.60)	<0.0001
	Ventricular extrasystole	70969(4.11)	16086(3.09)	<0.0001
	First-degree AVB	69092(4.00)	15283(2.93)	<0.0001
	RBBB	98430(5.70)	21888(4.20)	<0.0001
	LBBB	8974(0.52)	1990(0.38)	<0.0001
	Left anterior fascicular block	17405(1.01)	3784(0.73)	<0.0001
**Baseline (Jan 23, 2018 -Apr 7, 2018 and Jan 23, 2019 -Apr 7, 2019)**	Normal ECG	53708(42.65)	21525(46.24)	<0.001
	Sinus bradycardia	7332(5.82)	2779(5.97)	0.25
	Atrial extrasystole	9797(7.78)	2543(5.46)	<0.0001
	Ventricular extrasystole	6164(4.90)	1648(3.54)	<0.0001
	First-degree AVB	4632(3.68)	1170(2.51)	<0.0001
	RBBB	6819(5.42)	1959(4.21)	<0.0001
	LBBB	650(0.52)	183(0.39)	0.0011
	Left anterior fascicular block	1334(1.06)	414(0.89)	0.0018
**Baseline (Apr 8, 2018-Dec 31,2018 and Apr 8, 2019-Dec 31,2019)**	Normal ECG	421816(43.68)	104847(48.19)	<0.0001
	Sinus bradycardia	95825(9.92)	20276(9.32)	<0.0001
	Atrial extrasystole	63075(6.53)	9757(4.48)	<0.0001
	Ventricular extrasystole	36880(3.82)	6454(2.97)	<0.0001
	First-degree AVB	37259(3.86)	6068(2.79)	<0.0001
	RBBB	53823(5.57)	8850(4.07)	<0.0001
	LBBB	4826(0.50)	774(0.36)	<0.0001
	Left anterior fascicular block	9853(1.02)	1629(0.75)	<0.0001

**Lockdown (Jan 23, 2020 -Apr 7, 2020)**	Normal ECG	11144(35.93)	9063(40.77)	<0.0001
	Sinus bradycardia	1717(5.54)	1421(6.39)	<0.0001
	Atrial extrasystole	2701(8.71)	1376(6.19)	<0.0001
	Ventricular extrasystole	1874(6.04)	961(4.32)	<0.0001
	First-degree AVB	1548(4.99)	820(3.69)	<0.0001
	RBBB	1969(6.35)	1154(5.19)	<0.0001
	LBBB	216(0.70)	117(0.53)	0.014
	Left anterior fascicular block	335(1.08)	192(0.86)	0.013
**Post-lockdown era (Apr 8, 2020 -Dec 31, 2020)**	Normal ECG	203813(41.91)	83150(48.48)	<0.0001
	Sinus bradycardia	47026(9.67)	17770(10.36)	<0.0001
	Atrial extrasystole	32532(6.69)	6994(4.08)	<0.0001
	Ventricular extrasystole	19914(4.10)	4898(2.86)	<0.0001
	First-degree AVB	20570(4.23)	5498(3.21)	<0.0001
	RBBB	28819(5.93)	7178(4.19)	<0.0001
	LBBB	2683(0.55)	641(0.37)	<0.0001
	Left anterior fascicular block	4614(0.95)	1042(0.61)	<0.0001
**Post-SARS-CoV-2 era (Jan 23-Apr 7, 2021)**	Normal ECG	22910(40.18)	14779(49.01)	<0.0001
	Sinus bradycardia	3932(6.90)	2049(6.79)	0.57
	Atrial extrasystole	4158(7.29)	1406(4.66)	<0.0001
	Ventricular extrasystole	2835(4.97)	955(3.17)	<0.0001
	First-degree AVB	2536(4.45)	816(2.71)	<0.0001
	RBBB	3340(5.86)	1266(4.20)	<0.0001
	LBBB	293(0.51)	143(0.47)	0.43
	Left anterior fascicular block	583(1.02)	221(0.73)	<0.0001

The severe AVB includes second-degree type 2, high-degree and third-degree AVB.

SVT, supraventricular tachycardia; AVB, atrioventricular block; RBBB, right bundle branch block; LBBB, left bundle branch block.

Supplementary Table 12. Absolute numbers of the new COVID-19 cases or deaths.

	lockdown (Between Jan. 23 and Apr. 7, 2020)	post-lockdown (Between Apr. 8 and Dec. 31, 2020)	post-SARS-CoV-2 (Between Jan. 23 and Apr. 7, 2021)
new COVID-19 cases	81233	5270	1454
new COVID-19 deaths	3316	1301	1

Supplementary Figure 1. Medical visits with ECG examination (2016-2019).

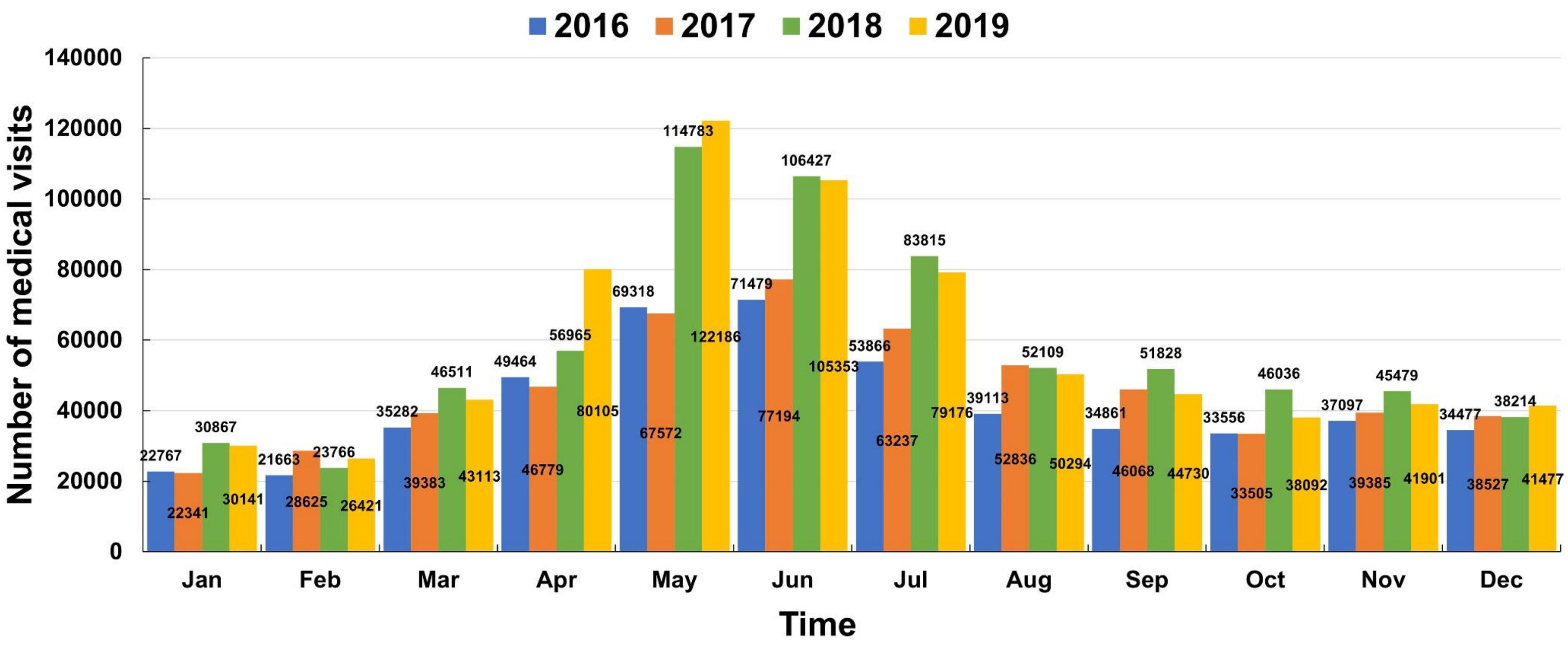

Supplementary Figure 2. Relationship between the number of new COVID-19 cases or deaths in China and the number of medical visits in the following 3-28 days during the lockdown in Shanghai.

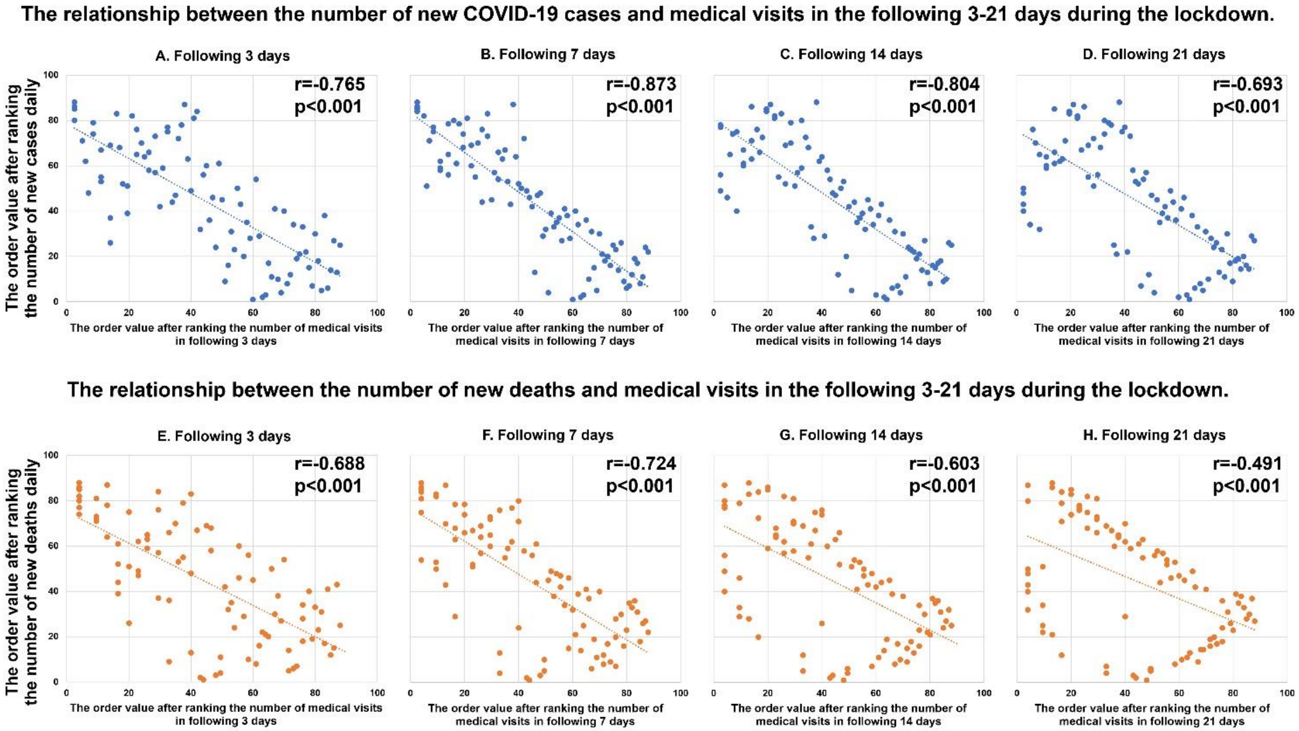

A-D. The relationship between the number of new COVID-19 cases and the number of medical visits in the following 3, 7, 14 and 28 days during the lockdown in Shanghai. **E-H.** The relationship between the number of new COVID-19 related deaths and the number of medical visits in the following 3, 7, 14 and 28 days during the lockdown in Shanghai. The horizontal axis represents the order value after ranking the number of daily new cases or deaths in China. The longitudinal axis represents the order value after ranking the number of daily medical visits in Shanghai.

Supplementary Figure 3. The relationship between the number of new COVID-19 cases or deaths in China and the number of medical visits during the following 3-14 days in Shanghai in the post-lockdown and post-SARS-CoV-2 eras (between Apr.8,2020 and Apr.7,2021).

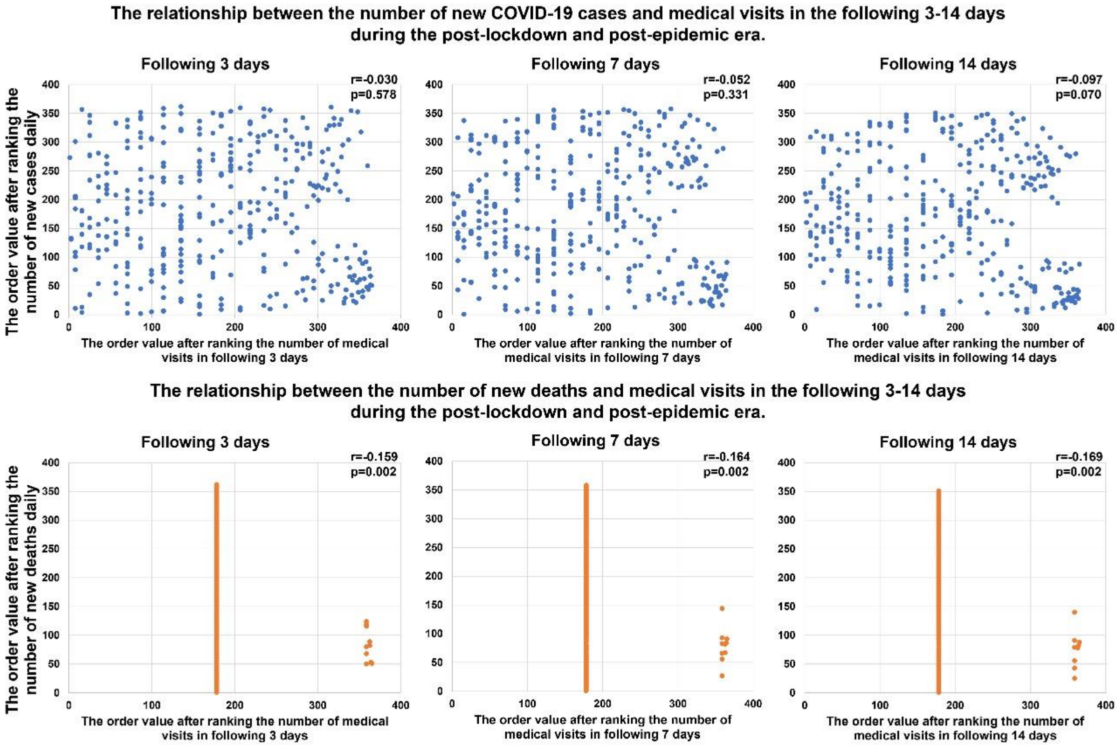

The horizontal axis represents the order value after ranking the number of daily new cases or deaths in China.

The longitudinal axis represents the order value after ranking the number of daily medical visits in Shanghai.

Supplementary Figure 4. Medical visits of patients with different age groups.

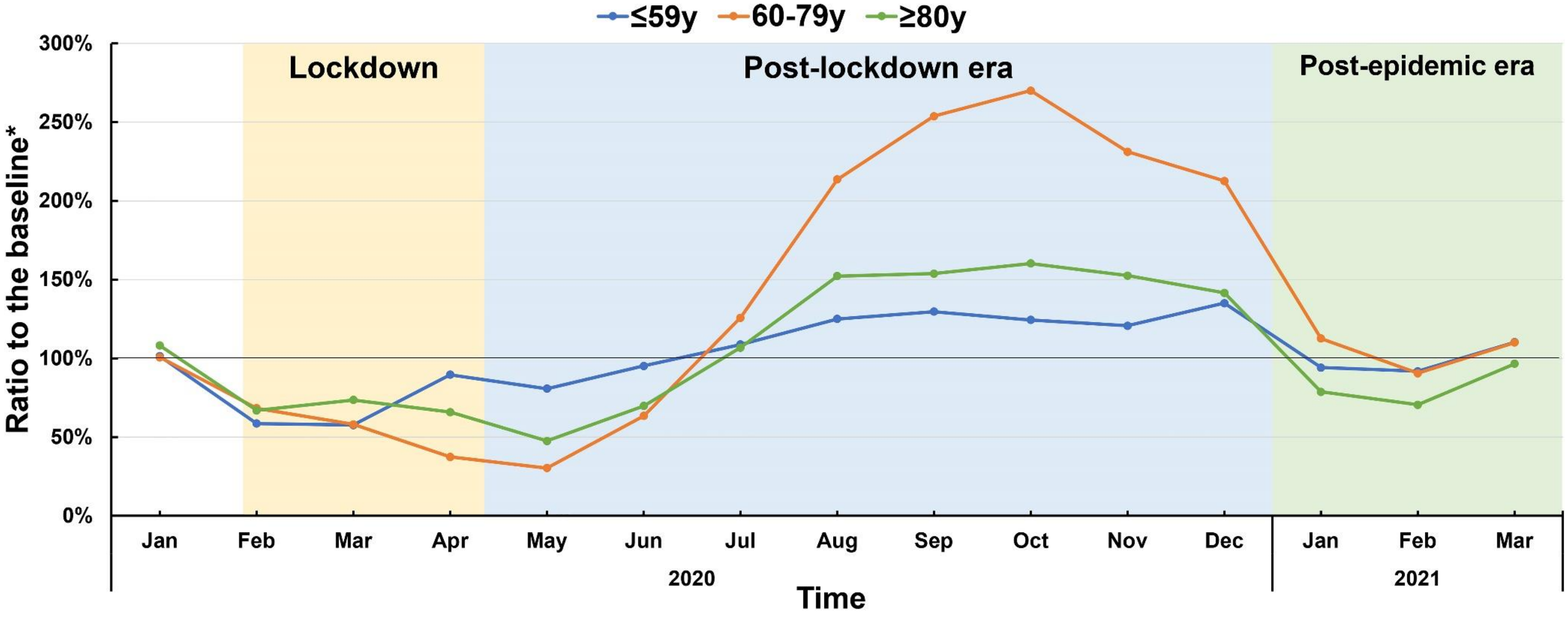

* The average number of 2018 and 2019.

Supplementary Figure 5. Medical visits of female and male patients.

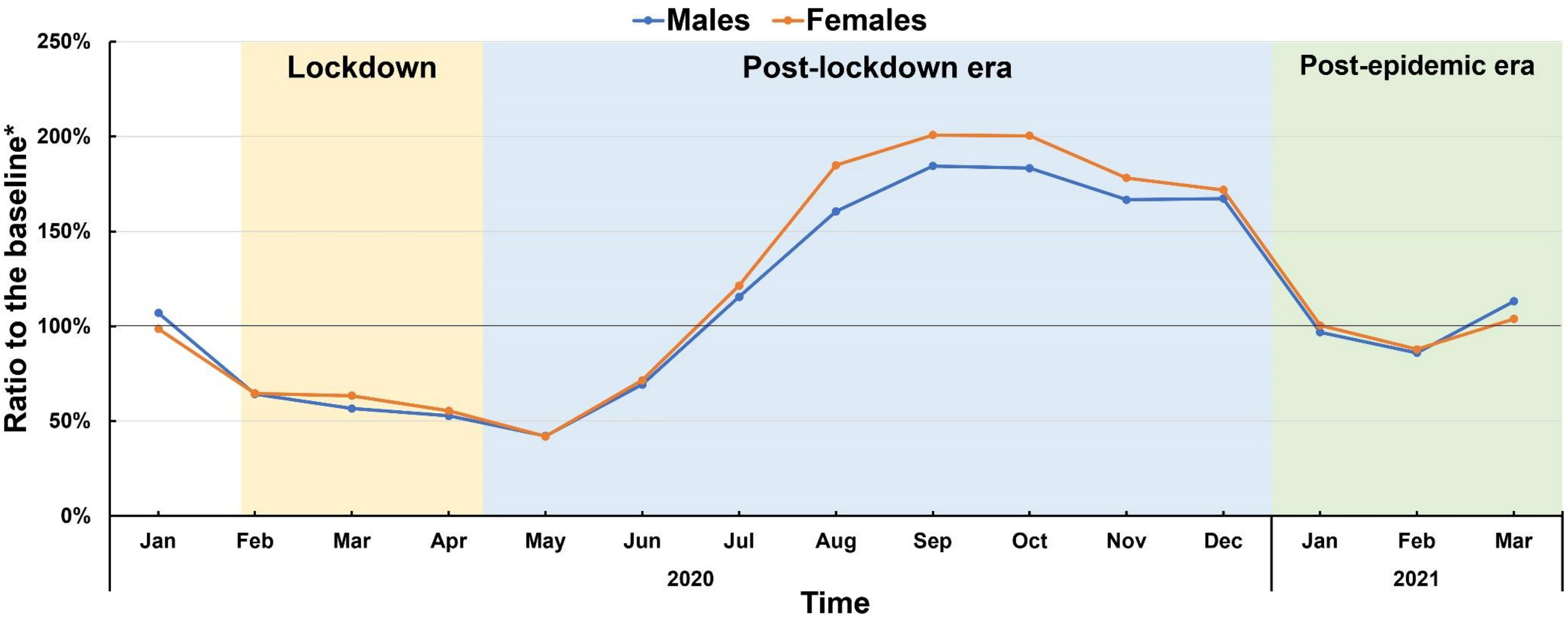

* The average number of 2018 and 2019.

Supplementary Figure 6. Medical visits of patients with cardiac arrhythmias exhibiting pattern 1 (stable-increase-stable) behavior.

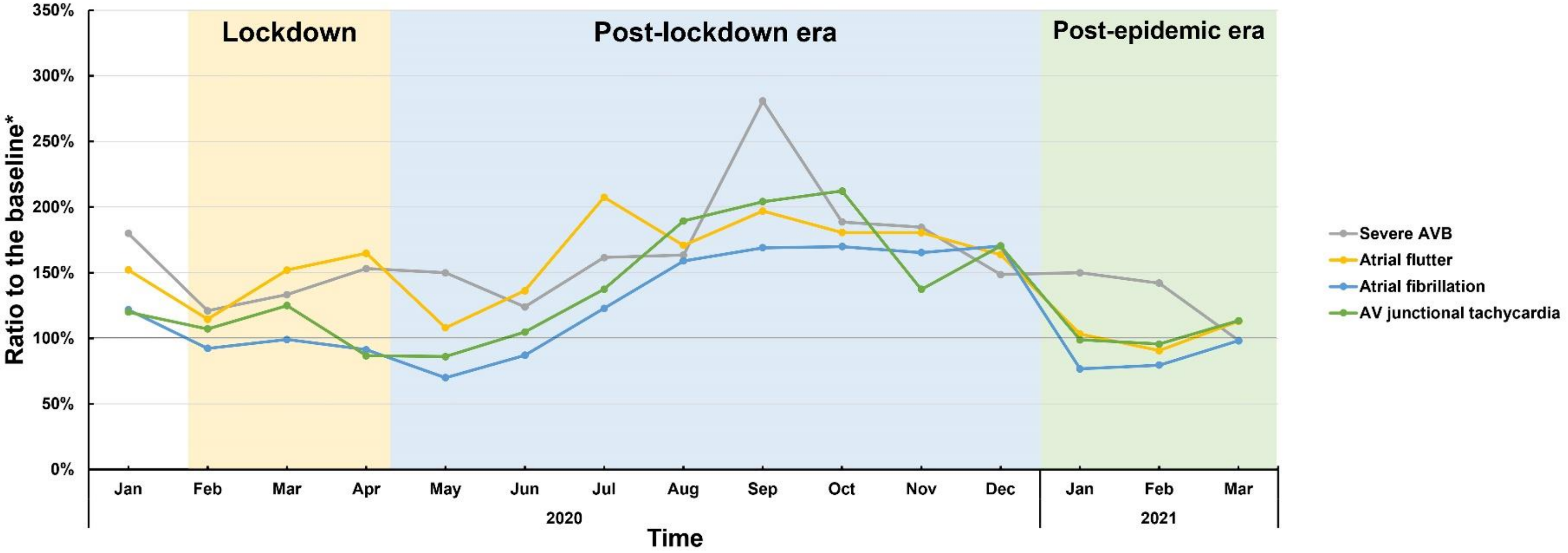

p value†	2020 vs baseline												2021 vs baseline			Lockdown	Post-lockdown	Post-pandemic
	Jan	Feb	Mar	Apr	May	Jun	Jul	Aug	Sep	Oct	Nov	Dec	Jan	Feb	Mar			
Severe AVB	0.52	0.63	0.57	0.25	0.25	0.39	0.19	0.037	0.00094	0.032	0.035	0.48	0.15	0.53	0.90	0.82	<0.0001	0.68
Atrial flutter	0.0074	0.44	0.0049	<0.0001	0.95	0.044	<0.0001	0.00056	<0.0001	0.0058	0.0024	0.0028	0.99	0.25	0.76	0.13	<0.0001	0.95
Atrial fibrillation	<0.0001	0.49	0.93	0.31	0.0028	0.14	0.024	0.00045	<0.0001	0.0022	0.00033	<0.0001	0.026	0.037	0.82	0.17	<0.0001	0.12
AV junctional tachycardia	0.00060	0.92	0.14	0.22	0.21	0.85	0.044	0.00041	<0.0001	0.0025	0.060	0.00028	0.79	0.55	0.49	0.79	<0.0001	0.80

* Average number of 2018 and 2019.

† The comparisons were obtained by Mann-Whitney test.

AV, atrioventricular; AVB, atrioventricular block.

The severe AVB includes second-degree type 2, high-degree and third-degree AVB.

Supplementary Figure 7. Medical visits of patients with cardiac arrhythmias exhibiting pattern 3 (decrease-rebound-fallback) behavior.

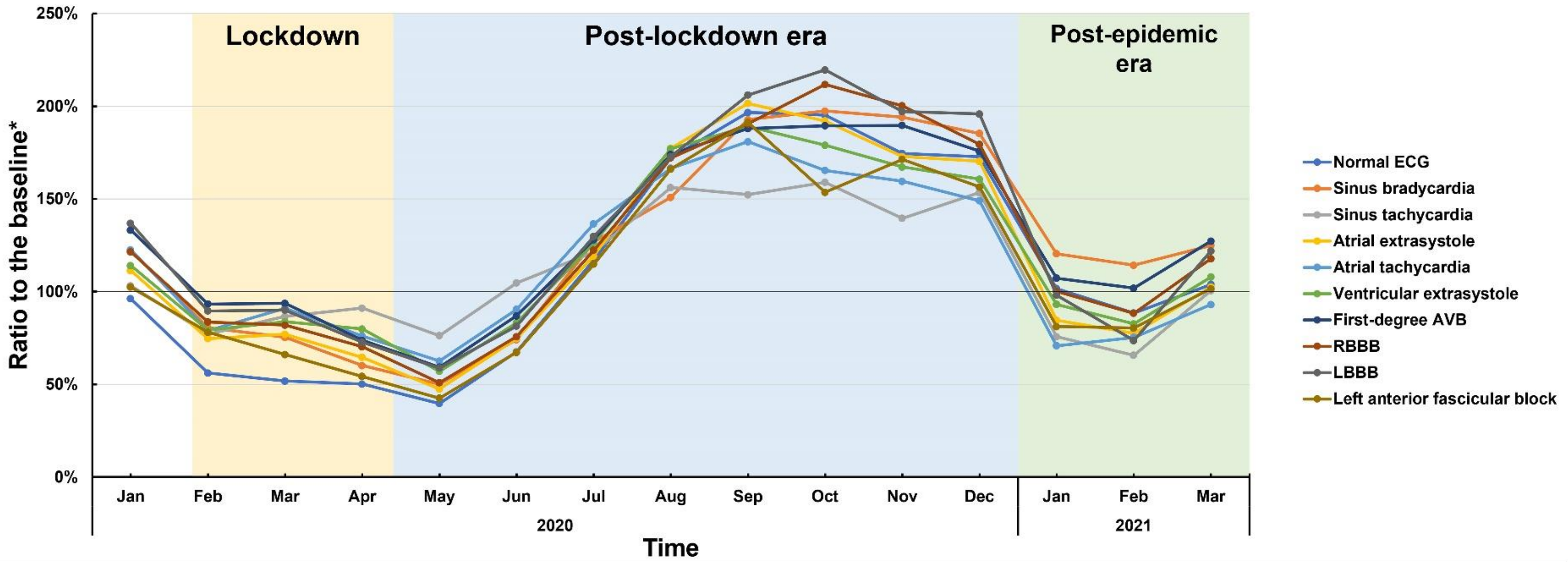

p value†	2020 vs Baseline												2021 vs Baseline				Post-	Post-
	Jan	Feb	Mar	Apr	May	Jun	Jul	Aug	Sep	Oct	Nov	Dec	Jan	Feb	Mar	Lockdown	lockdown	pandemic
Normal ECG	0·76	<0·0001	<0·0001	<0·0001	<0·0001	0·0010	0·064	0·00034	<0·0001	0·0052	0·00027	<0·0001	0·55	0·62	0·59	<0·0001	0·015	0·96
Sinus tachycardia	<0·0001	0·0010	0·038	0·26	0·00096	0·52	0·023	0·00033	<0·0001	0·010	0·00071	<0·0001	0·00029	<0·0001	0·83	<0·0001	<0·0001	0·0020
Atrial extrasystole	<0·0001	0·0018	0·0026	0·00031	<0·0001	0·0087	0·065	0·00051	<0·0001	0·0057	0·00083	<0·0001	0·16	0·036	0·64	<0·0001	0·00044	0·20
Atrial tachycardia	0·00061	0·15	0·32	0·055	0·00014	0·30	0·013	0·00049	<0·0001	0·023	0·0035	0·00094	0·0083	0·051	0·34	0·0041	<0·0001	0·016
Ventricular extrasystole	<0·0001	0·040	0·092	0·039	<0·0001	0·078	0·0081	0·00032	<0·0001	0·0060	0·00056	<0·0001	0·62	0·18	0·25	0·0036	<0·0001	0·79
First-degree AVB	0·0014	0·70	0·51	0·0083	0·00071	0·20	0·028	0·00052	<0·0001	0·0067	0·00042	0·00020	0·63	0·91	0·0052	0·13	<0·0001	0·18
RBBB	<0·0001	0·065	0·019	0·0023	<0·0001	0·016	0·019	0·00036	<0·0001	0·0016	<0·0001	<0·0001	0·69	0·34	0·056	0·0044	<0·0001	0·81
LBBB	0·0073	0·24	0·37	0·014	0·00010	0·075	0·022	0·0018	<0·0001	0·00057	<0·0001	<0·0001	0·52	0·032	0·098	0·039	<0·0001	0·53
Left anterior fascicular block	<0·0001	0·030	0·0012	0·00032	<0·0001	0·0019	0·15	0·0047	<0·0001	0·074	0·00024	0·0042	0·083	0·081	0·90	<0·0001	0·18	0·17

* The average number of 2018 and 2019.

† The comparisons were obtained by Mann-Whitney test.

AVB, atrioventricular block; RBBB, right bundle branch block; LBBB, left bundle branch block.

Supplementary Figure 8. Relationship between the number of new COVID-19 cases or deaths and the number of medical visits of specific cardiac arrhythmias in the following 7 days during the lockdown in Shanghai.

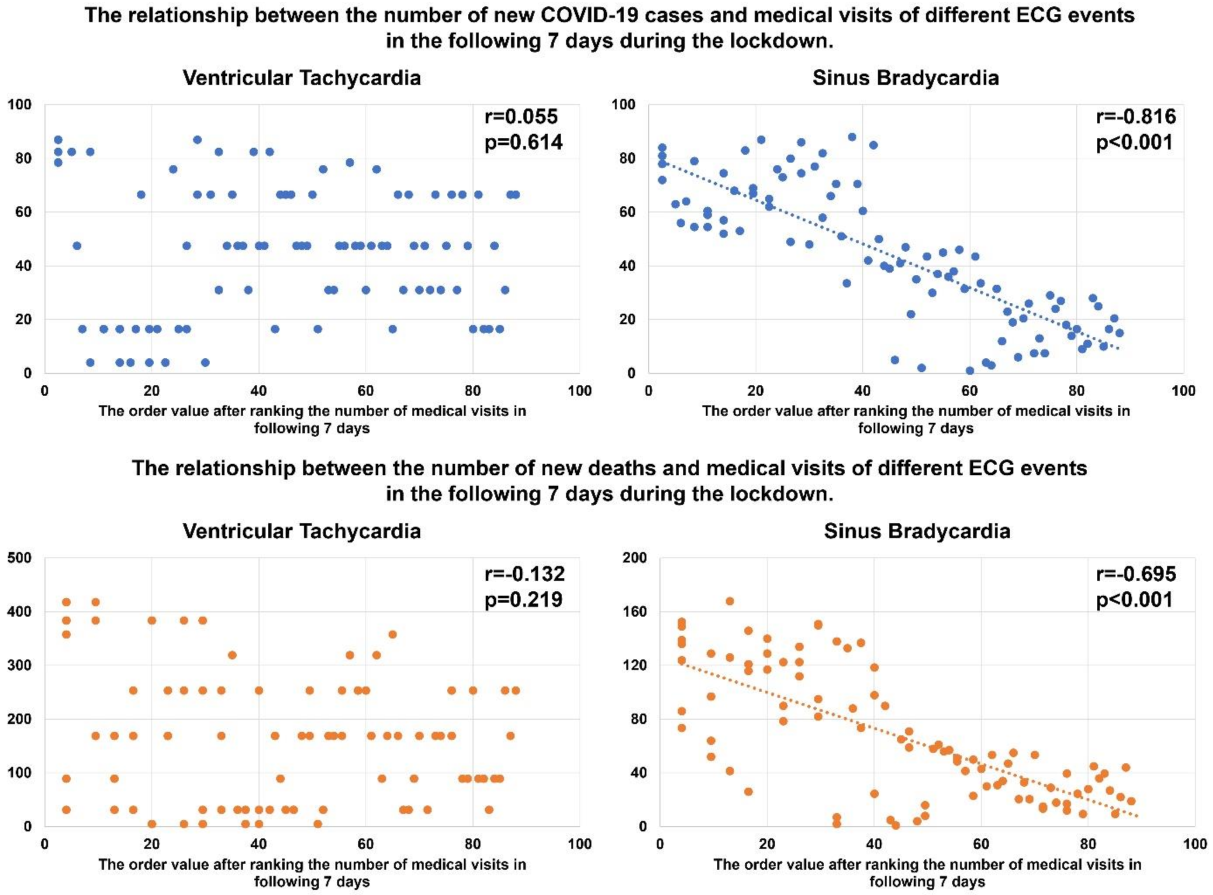

The horizontal axis represents the order value after ranking the number of daily new COVID-19 cases or new deaths. The longitudinal axis represents the order value after ranking the number of daily medical visits of specific cardiac arrhythmias in Shanghai.

Supplementary Figure 9. The relationship between the number of new COVID-19 cases or deaths in Shanghai and the number of medical visits in Shanghai in the following 3-14 days during the lockdown.

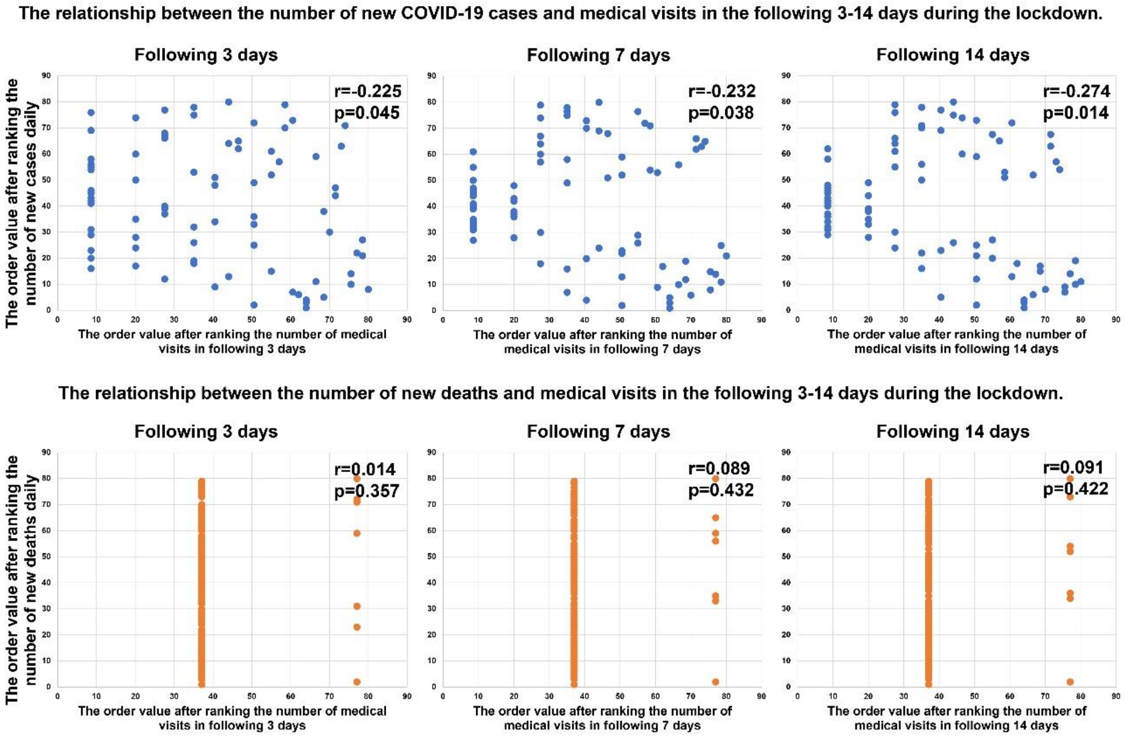

The horizontal axis represents the order value after ranking the number of daily new COVID-19 cases or deaths in Shanghai. The longitudinal axis represents the order value after ranking the number of daily medical visits in Shanghai.
